# Prevalence of physical and sexual violence and psychological abuse among adolescents and young adults living with HIV in Zambia

**DOI:** 10.1371/journal.pone.0235203

**Published:** 2020-06-25

**Authors:** Katherine G. Merrill, Jacquelyn C. Campbell, Michele R. Decker, John McGready, Virginia M. Burke, Jonathan K. Mwansa, Sam Miti, Christiana Frimpong, Caitlin E. Kennedy, Julie A. Denison

**Affiliations:** 1 Department of International Health, Johns Hopkins Bloomberg School of Public Health, Baltimore, Maryland, United States of America; 2 Department of Community-Public Health, Johns Hopkins School of Nursing, Baltimore, Maryland, United States of America; 3 Department of Population, Family & Reproductive Health, Johns Hopkins Bloomberg School of Public Health, Baltimore, Maryland, United States of America; 4 Department of Biostatistics, Johns Hopkins Bloomberg School of Public Health, Baltimore, Maryland, United States of America; 5 Arthur Davison Children’s Hospital, Ndola, Zambia; RTI International, UNITED STATES

## Abstract

**Background:**

Little is known about violence against HIV-positive adolescents and young adults (AYA) in sub-Saharan Africa. This analysis examines experiences of violence victimization, and the perpetrators of this violence, among AYA living with HIV, aged 15–24 years, in Zambia.

**Methods:**

We analyzed baseline data from 272 AYA (60.1% female, 71.0% perinatally infected) enrolled in Project YES! (Youth Engaging for Success), a randomized controlled trial conducted in four HIV clinics in Ndola, Zambia. Violence measures were adapted from the ICAST-C and the WHO Multi-Country Study on Women’s Health and Domestic Violence. Youth could report up to 12 perpetrator types for past-year experiences of violence. We estimated lifetime and past-year prevalence of physical violence, psychological abuse, and forced sex, disaggregated by sex and age group. Estimates were weighted using sex and age data from the 2013–14 Zambian Demographic and Health Survey to be representative of HIV-positive AYA in Zambia.

**Results:**

Estimated lifetime prevalence of any violence victimization was 78.2%. Past-year prevalence was 72.0% among males and 74.5% among females. Almost half of AYA (46.1%) had ever experienced polyvictimization (2+ types of violence). Psychological abuse was most common (70.4% lifetime, 65.3% past-year), followed by physical violence (50.8% lifetime, 44.7% past-year) and forced sex (10.4% lifetime, 4.7% past-year). Among past-year victims, males experienced more violence than females from a friend/peer (74.3% vs. 45.1%, p<0.001); females experienced more violence than males from a romantic partner (33.3% vs. 5.0%, p<0.001), parent/caregiver (32.4% vs. 17.6%, p = 0.02), and stranger (19.7% vs. 5.2%, p<0.001).

**Conclusion:**

The widespread and overlapping prevalence of multiple types of violence highlights the critical need for prevention and response efforts that are tailored to youths’ sex and the perpetrator type. Future research should explore violence victimization and HIV outcomes, and the measurement of psychological abuse and sexual violence, among HIV-positive AYA in the region.

## Introduction

In many settings in sub-Saharan Africa (SSA), adolescents and young adults (AYA) face high levels of violence victimization. A systematic review found that over 50% of adolescents (aged 15–17 years) from 24 African countries had experienced physical, sexual, or emotional violence, or bullying, in the past year [1]. In a meta-analysis, roughly one-third to one-half of young women (aged 20–24 years) reported having ever experienced physical or sexual intimate partner violence across 8 countries in Eastern or Southern Africa [2]. Literature from high-income settings and SSA alike has demonstrated that young people who are exposed to violence from a range of perpetrator types in home, school, and community settings, are at risk of negative health outcomes in the short- and long-term, including greater likelihood of depression [2–4], substance use [2, 3, 5, 6], suicidal ideation [2, 3, 7], anti-social behavior [8], and risky sexual behavior [2, 4].

Studies have identified intimate partner violence (IPV) as an important concern among HIV-positive adult women in SSA [9, 10]. These studies have demonstrated that HIV-positive women have a higher risk of IPV than their HIV-negative counterparts [11] and that experiencing violence can disrupt antiretroviral therapy (ART) adherence and prevent viral suppression [12]. Moreover, for adult women, violence or the fear of violence—particularly from intimate partners—has been associated with increased sexual risk behavior [13] and HIV non-disclosure [14], additional barriers to the prevention of HIV transmission.

However, comparatively little attention has been paid to violence from any perpetrator against AYA living with HIV in the region. This is despite research showing that AYA are undergoing cognitive, psychosocial, emotional, and social changes [15] and therefore have different needs than adults. SSA is home to the majority of the world’s HIV-positive youth (84%, 1.7 million) [16], and three in four new HIV infections among 15-19-year-olds occur in SSA [17]. Some studies—for example, in Tanzania [18], South Africa [19, 20], and Malawi [21]—have assessed exposure to violence among HIV-positive youth as an independent or adjustment variable, reporting wide-ranging prevalence figures derived from widely varying measures and methodologies. Only one study was identified for which violence was the primary focus in a population of HIV-positive adolescents in SSA; this study by Cluver et al. found that between 41% and 47% of the sample of 1,060 South African boys and girls (ages 10–19) reported exposure to past-year physical or verbal violence from teachers, peers, or community members [22] but did not measure IPV. While there is limited data on HIV-positive adolescents (up to age 19), there is virtually no information available on HIV-positive young men (aged 20–24 years) and violence victimization in SSA. This paucity of data is concerning since the forms and perpetrators of violence often differ for male and female youth; males, for instance, tend to be at higher risk of male-on-male interpersonal violence [23] and females of dating and sexual violence from men [23, 24].

It is critical to ascertain the magnitude and perpetrators of violence against HIV-positive youth in SSA to inform the development of appropriate prevention and response efforts. Such efforts could impede the negative health and developmental consequences of violence, and also prevent HIV disease progression and reduce the onward transmission of HIV. Understanding the epidemiology of violence against both male and female AYA living with HIV is particularly needed in Zambia, which has among the highest prevalence of both HIV (12% among adults, [25]) and partner violence (47% among ever-married women, [26]) globally. Failure to recognize the role of violence in the lives of HIV-positive AYA could ultimately hamper global efforts to end the AIDS epidemic by 2030 [27]. To address this gap, the current study describes the prevalence of physical violence, psychological abuse, and sexual violence from multiple perpetrator types against AYA living with HIV in Zambia.

## Materials and methods

### Design and procedures

We conducted a cross-sectional analysis using baseline data from the Project YES! (Youth Engaging for Success) randomized controlled trial (RCT) [28, 29]. The trial was designed to assess the impact of a peer mentoring intervention on viral load and other HIV-related outcomes among AYA living with HIV in Ndola, Zambia. Ndola, the capital of the Copperbelt Province, is a peri-urban community with a population of about 370,000, [30] located near the Democratic Republic of Congo border.

Baseline data were collected from December 2017 through May 2018 in four purposively-selected HIV clinics, including a children’s hospital, an adult hospital, and two primary health facilities. AYA were consecutively recruited if they were aged 15–24 years, spoke English or Bemba, knew their HIV status, were on ART for at least six months, and were available for study activities over 18 months. AYA were ineligible if they were too sick to participate as determined by the recruiting healthcare provider, were attending boarding school, had a sibling already enrolled in the study, or had participated in a recent adolescent/caregiver intervention study held at two of the study clinics.

Potentially eligible patients were approached by a health care provider and referred, if interested, to a trained research staff member who explained the study, screened for eligibility, and conducted informed consent. In line with Zambian law, written parental/caregiver consent and youth assent was required for participants aged 15–17 years [31]. Research staff members administered baseline surveys to all consenting and assenting youth participants, in either Bemba or English, during face-to-face interviews using Magpi software on tablet computers. Given that baseline surveys included questions about experiences of violence and suicide ideation, in addition to the potential for sensitive issues to arise during peer mentoring meetings, the team developed and implemented a safety protocol with referral procedures for both peer mentors and data collectors to connect youth participants with health care providers (HCP) for additional care (see further description under Ethics). Data were uploaded to a secure server and checked for quality. Additionally, participants’ ART start dates were abstracted from their medical charts.

### Measures

Self-reported measures of violence victimization were adapted from the internationally-recognized and widely-used IPSCAN Child Abuse Screening Tool (ICAST) [32] and the World Health Organization (WHO) Multi-Country Study on Women’s Health and Domestic Violence against Women (WHO MCS) [33]. The ICAST-C (Child Version) is shown to have good internal consistency and construct validity [34] and has been administered in numerous settings in SSA, including Mali, Uganda, and Zambia [35]. Given that the ICAST was designed for children ages 11 to 18, it was supplemented by items from the WHO MCS, which has been widely used to measure violence from intimate partners across the region. The WHO MCS items are similar to those items used to assess violence in the 2013–14 Zambia Demographic and Health Survey (DHS) [26].

**Fig 1:**
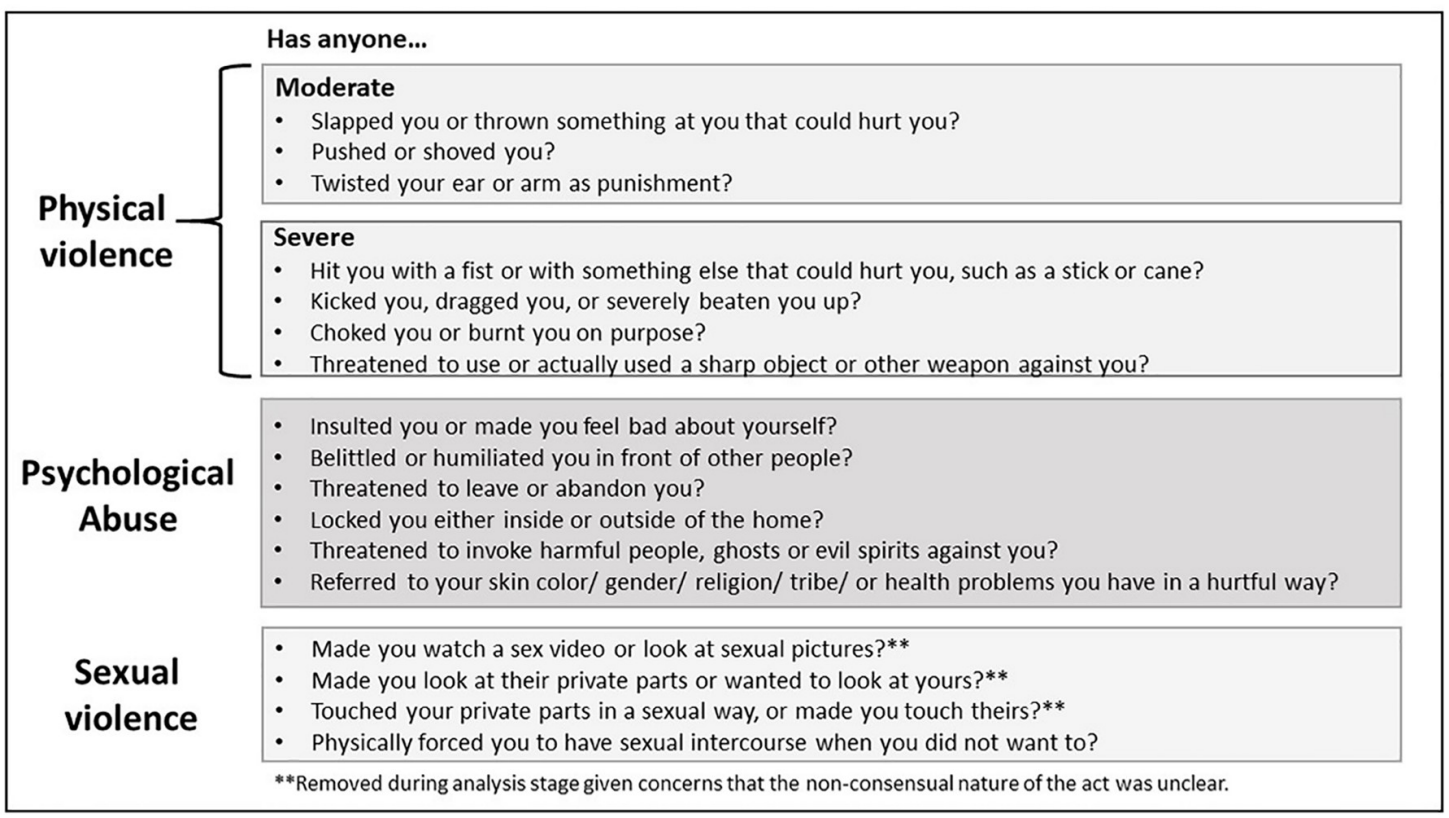
Measures of violence victimization, derived from the ICAST Child Abuse Screening Tool and the WHO Multi-Country Study on Women’s Health and Domestic Violence.

Surveys queried acts of physical (7 items), psychological (6 items), and sexual (4 items) violence victimization (Fig 1). Participants were asked if they had ever experienced the act (yes/no), and if yes, how often they had experienced it in the past year: never, once, a few times, or many times. They were classified as having experienced any of the three types of victimization if reporting at least one of the associated acts. Experiences of physical violence were distinguished by severity level based on WHO guidelines [33], with three items capturing ‘moderate’ violence (e.g. pushed/shoved) and four items ‘severe’ violence (e.g. choked/burnt). A single variable of forced sex from the WHO MCS was used to measure sexual violence. The remaining three items from the ICAST were not analyzed since they described sexual behaviors that may have been interpreted as consensual sexual experiences by older youth with intimate partners (Fig 1). The age of first experience of forced sex was also measured. We found adequate reliability for the measures of physical violence and psychological abuse, with Cronbach’s alphas ranging between 0.61 and 0.66 [36]. Participants were classified as having experienced polyvictimization if they reported two or more types of victimization (physical, psychological, or sexual), ever or in the past year. Finally, for past-year measures, participants were asked who perpetrated the act and could choose all that apply from 12 possible perpetrator types: romantic partner, parent/caregiver, other family member, friend or peer, stranger, school staff member, employer, health care worker, neighbor, religious leader, military/police, or someone else the youth knows. Survey items were reviewed by Zambian coauthors to ensure their appropriateness. The full instrument was translated into Bemba and pilot-tested among youth in Ndola for comprehension and clarity.

Socio-demographic characteristics measured in the baseline survey included the youth’s age (categorized as 15-19/20-24 years), sex (male/female), completion of primary school (did/did not complete), current employment status (employed/not), and orphanhood status (none/single orphan/double orphan). HIV measures included self-reported mode of HIV acquisition (from parents/through sex/another way/don’t know or refused) and length of time on ART (categorized as 6 months to <3 years, 3 to <6 years, or 6+ years, based on the dates the participant began ART and enrolled in the study).

### Analyses

Descriptive analyses were conducted to observe distributions of study variables. Lifetime and past-year prevalence of physical violence, psychological abuse, and forced sex was estimated using percentages and 95% confidence intervals. Prevalence figures were disaggregated by sex given recognized differences in experiences of violence for male compared with female youth [23, 24]. Past-year prevalence was further disaggregated by youth’s sex and age group (15–19 and 20–24) since experiences of violence may differ for younger compared to older youth given their unique developmental stages [15] and to facilitate comparisons with other international datasets, which have used these age groups [26, 37]. To explore whether experiences of violence differ based on the youth’s self-reported mode of HIV acquisition, we compared the prevalence of past-year violence (all types) for those reporting acquiring HIV from their parents to those who reported any other mode of acquisition. We further summarized past-year prevalence according to perpetrator types. Venn diagrams were generated to visually depict respondents’ overlapping experiences of physical violence, psychological abuse, and/or forced sex victimization in their lifetimes and in the past year.

Prevalence estimates were weighted for age (15–19 and 20–24 years) and sex (male and female) using the Zambian 2013–14 DHS [26] to be representative of HIV-positive males and females, ages 15–24 years, in Zambia. We used Zambia DHS data to derive the number and proportion of HIV-positive individuals within the following four categories: males ages 15–19 years; females ages 15–19 years; males ages 20–24 years; and females ages 20–24 years. We then divided the DHS proportion by the proportion in our sample to derive a post-stratification weight for each of category that we subsequently applied to our population estimates (Table 1). Reported sample sizes and percentages are all weighted. Differences in proportions were assessed using F tests to accommodate the complex sampling design resulting from the use of sampling weights.

**Table 1:** Application of post-stratification weights, using the 2013–14 Zambia Demographic and Health Survey (DHS).

	Population N from Zambia DHS	Proportion of population N from Zambia DHS	Sample n	Proportion of sample n	Weight
Males, ages 15–19	133 (3,246*0.041)	17.39%	83	30.51%	**0.5700** (.1739/0.3051)
Females, ages 15–19	157 (3,273*0.048)	20.52%	90	33.09%	**0.6201** (.2052/0.3309)
Males, ages 20–24	168 (2,307*0.073)	21.96%	28	10.29%	**2.1341** (.2196/0.1029)
Females, ages 20–24	307 (2,745* 0.112)	40.13%	71	26.10%	**1.538** (.4013/0.2610)
Total	765		272		

While a common threshold for physical or sexual violence is one or more acts of violence, researchers have questioned whether this same threshold should apply for psychological abuse, which has more variation in form and acceptability across cultures [38]. A single act of being insulted, for instance, is thought by many to be too low a threshold to constitute psychological abuse [39]. Hence, we conducted a sensitivity analysis to assess whether using a stricter definition of past-year psychological abuse would affect the research results. Participants were classified as victims if they reported experiencing two or more acts of psychological abuse from at least one type of perpetrator in the past year. Analyses were carried out using STATA 14 [40].

### Ethical considerations

Informed consent was obtained from each participant by a study team member in a private space in or near the clinic. Drawing on WHO ethical and safety recommendations [41], caregiver consent forms used broad terms to describe the research (e.g. health, safety) to protect minors who may be experiencing violence from the caregiver; youth consent forms included more details about the surveys. Research staff were trained on the ethics of violence research. To minimize under-reporting, violence questions were preceded by less sensitive topics. Introductory text was intended to help participants feel comfortable disclosing their experiences and avoid interpreting questions as judgmental or stigmatizing [41]. Based on the safety protocol, participants were referred to a HCP at each clinic if reporting severe past-year physical violence, lifetime sexual violence, or past-week suicidal thoughts. HCPs responded according to clinical practice, local policy, and Zambian law. The Johns Hopkins Bloomberg School of Public Health Review Board and the ERES Converge ethical review board in Zambia, along with the Zambia Ministry of Health through the National Health Research Authority, reviewed and approved the research.

## Results

### Sample characteristics

Of 373 youth approached, 276 (74%) enrolled, of whom data were analyzed for 272. Three were excluded during data cleaning for not meeting the inclusion criteria of being on ART for at least six months and one baseline survey was missing from the database. In the weighted sample, about two-thirds were female (60.1%) and about two-thirds were aged 20–24 years (61.8%) (Table 2). Most had completed primary school (89.1%), and about 11% were currently employed at the time of the survey. Three-quarters reported having lost at least one parent (75.5%) and a similar percent reported having acquired HIV from their parents (71.0%). Almost two-thirds (61.4%) had been on ART for six or more years.

**Table 2:** Sample characteristics of adolescents and young adults living with HIV in Ndola, Zambia, stratified by sex (n = 272).

	Total	Male	Female	p value
	272 (100%)	108 (39.9%)	163 (60.1%)	//
Age				
15–19	104 (38.2%)	48 (44.2%)	56 (34.2%)	0.12
20–24	168 (61.8%)	60 (55.8%)	107 (65.8%)	
Completed primary school (n = 271)				
Completed	242 (89.1%)	104 (96.3%)	137 (84.3%)	<0.001
Did not complete	30 (10.9%)	4 (3.7%)	25 (15.7%)	
Currently employed				
No	241 (88.9%)	98 (91.0%)	143 (87.5%)	0.50
Yes	30 (11.1%)	10 (9.0%)	20 (12.4%)	
Orphanhood				
None	67 (24.6%)	20 (18.8%)	46 (28.4%)	0.26
Single orphanhood	105 (38.9%)	43 (40.2%)	62 (38.0%)	
Double orphanhood	99 (36.6%)	44 (41.1%)	55 (33.6%)	
Mode of HIV acquisition				
From parents	193 (71.0%)	88 (80.9%)	105 (64.4%)	<0.001
Through sex	34 (12.4%)	1 (0.5%)	33 (20.0%)	
Another way	14 (5.0%)	6 (5.2%)	8 (4.9%)	
Don't know/refused	32 (11.6%)	15 (13.4%)	17 (10.5%)	
Time on ART treatment (n = 269)				
6 months to <3 years	66 (24.2%)	13 (11.8%)	53 (32.4%)	<0.001
3 to <6 years	37 (13.7%)	10 (8.9%)	28 (16.9%)	
6+ years	167 (61.4%)	85 (78.7%)	81 (50.0%)	

All n's and percentages are weighted for age (15–19 and 20–24 years) and sex (male and female) using the Zambian 2013–14 DHS [26]; % are column percentages. P values are from F tests.

### Prevalence of violence victimization

The estimated prevalence of violence victimization (physical violence, psychological abuse, or forced sex) among HIV-positive AYA in Zambia was 78.2% for lifetime reports and 73.5% for past-year reports (Table 3). Across both timeframes assessed, psychological abuse was most common (70.4% lifetime, 65.3% past-year), followed by physical violence (50.8% lifetime, 44.7% past-year), followed by forced sex (10.5% lifetime, 4.7% past-year). Among victims of physical violence, over one third (36.9% lifetime, 34.0% past-year) experienced severe physical violence. There was weak evidence that females experienced almost twice as much severe physical violence in the past year than males (41.0% vs. 22.8%, p<0.07). Among lifetime victims of forced sex, the age at the first experience of forced sex ranged from 15 to 21 years for males (mean = 16.8 years, SD = 1.8) and 4 to 24 years for females (mean = 16.3 years, SD = 4.8) (p = 0.44 for males vs. females).

**Table 3:** Estimated lifetime and past-year prevalence of violence victimization among adolescents and young adults living with HIV in Zambia (n = 272).

	Lifetime % (95%CI)	Past year % (95%CI)	Past year, stratified by sex		
			Male (n = 108) % (95%CI)	Female (n = 163) % (95%CI)	p value
**Any victimization**					
Physical, psychological, or forced sex	78.2 (72.0, 83.4)	73.5 (67.0, 79.1)	72.0 (60.3, 81.2)	74.5 (66.6, 81.1)	0.69
**Type of victimization**					
Physical violence^	50.8 (43.9, 57.5)	44.7 (38.1, 51.5)	43.2 (32.5, 54.7)	45.7 (37.4, 54.1)	0.73
Moderate physical violence	95.6 (90.3, 98.1)	95.5 (89.5, 98.2)	97.5 (90.3, 99.4)	94.3 (84.5, 98.0)	0.33
Severe physical violence	36.9 (28.4, 46.4)	34.0 (25.3, 43.9)	22.8 (11.9, 39.1)	41.0 (29.6, 53.5)	0.07
Psychological abuse	70.4 (63.9, 76.2)	65.3 (58.6, 71.5)	65.3 (53.6, 75.4)	65.3 (57.0, 72.8)	0.99
Forced sex	10.5 (7.0, 15.5)	4.7 (2.6, 8.4)	4.1 (1.4, 11.5)	5.1 (2.5, 10.1)	0.75
Polyvictimization*					
No violence	21.8 (16.6, 28.0)	26.5 (20.9, 33.0)	28.1 (18.8, 39.7)	25.5 (18.9, 33.4)	0.84
1 type of violence	32.2 (26.2, 38.8)	35.7 (29.4, 42.6)	33.4 (23.6, 44.8)	37.3 (29.4, 45.9)	
2+ types of violence	46.1 (39.4, 52.9)	37.8 (31.4, 44.5)	38.6 (28.3, 50.0)	37.2 (29.5, 45.7)	

All n’s and percentages are weighted; %s are column percentages and may not add up to 100, since participants could select more than one form of violence. p values are from F tests.

^Percentages for moderate and severe physical violence are among those reporting any physical violence (n = 212 lifetime, n = 199 past-year)

*Categories are mutually exclusive.

**Fig 2:**
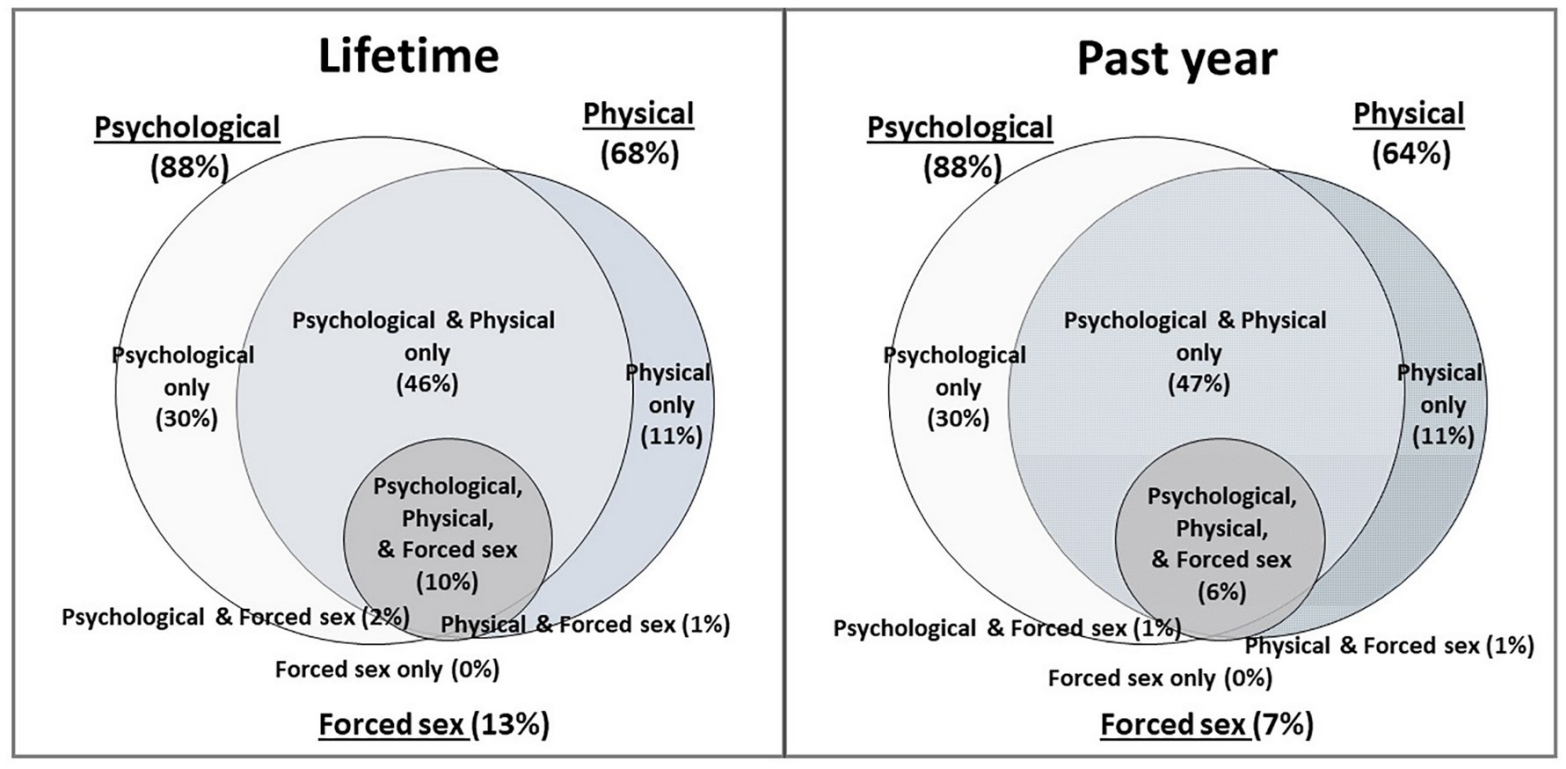
Overlapping experiences of physical violence, psychological abuse, and forced sex among HIV-positive adolescents and young adults reporting violence victimization, ever (weighted n = 212) and in the past year (weighted n = 199).

Almost half of HIV-positive AYA experienced polyvictimization in their lifetimes (46.1%), and over a third in the past year (37.8%) (Table 3). Heavy overlap was observed across all three forms of violence, and especially for psychological abuse and physical violence (Fig 2). Experience of forced sex was always accompanied with physical or psychological violence, for lifetime and past-year timeframes.

The prevalence or type of violence experienced did not significantly differ between males and females, including forced sex (past-year prevalence: 4.1% for males and 5.1% for females, p = 0.75). Among AYA who experienced past-year physical violence, females experienced more severe physical violence (41.0% vs. 22.8% among males) but this difference did not reach statistical significance (p = 0.07) (Table 3). For specific acts, females had a higher frequency of reports of being ‘kicked, dragged, or severely beaten up’ (p<0.05) and ‘locked inside or outside the home’ (p<0.001) than males in the past year (S1 Table).

When we disaggregated prevalence and perpetrators by participant age (15–19, and 20–24 years) and sex (Table 4), we found that among AYA ages 15–19, males compared to females experienced significantly higher levels of past-year psychological abuse (71.1% vs. 55.6%, p<0.05). Across both age groups, males experienced more friend/peer violence than females, but the difference only reached significance for AYA ages 15–19 years (57.8% vs. 33.3%, p<0.01).

**Table 4:** Estimated prevalence of past-year violence against adolescents and young adults living with HIV in Zambia, stratified by age group and sex (n = 272).

	Total % (95%CI)	Ages 15–19 (n = 104)			Ages 20–24 (n = 168)		
		Male (n = 48) % (95%CI)	Female (n = 56) % (95%CI)	p value	Male (n = 60) % (95%CI)	Female (n = 107) % (95%CI)	p value
**Any victimization**							
Physical, psychological, or forced sex	73.5 (67.0, 79.1)	77.1 (66.7, 33.2)	68.9 (58.5, 77.6)	0.23	67.9 (48.5, 82.6)	77.5 (66.1, 85.8)	0.33
**Type of victimization**							
Physical violence	44.7 (38.1, 51.5)	48.2 (37.6, 59.0)	52.2 (41.9, 62.4)	0.60	39.3 (23.0, 58.3)	42.3 (31.2, 54.2)	0.79
Psychological abuse	65.3 (58.6, 71.5)	71.1 (60.4, 79.9)	55.6 (45.1, 65.5)	0.04	60.7 (41.7, 77.0)	70.4 (58.6, 80.0)	0.36
Forced sex	4.7 (2.6, 8.4)	4.8 (1.8, 12.3)	6.7 (3.0, 14.2)	0.60	3.6 (0.5, 22.0)	4.2 (1.3, 12.5)	0.88
**Any victimization by perpetrator type**							
Romantic partner	16.3 (11.8, 22.1)	3.6 (1.2, 10.7)	15.6 (9.4, 24.7)	0.009	3.6 (0.5, 22.0)	29.6 (20.0, 41.4)	0.007
Parent/caregiver	20.4 (15.7, 26.0)	24.1 (16.0, 34.5)	30.0 (21.4, 40.3)	0.39	7.1 (1.8, 25.0)	21.1 (13.0, 32.4)	0.10
Other family member	31.0 (25.1, 37.7)	30.1 (21.2, 40.9)	33.3 (24.3, 43.8)	0.65	32.1 (17.5, 51.5)	29.6 (20.0, 41.4)	0.80
Friend/peer	41.6 (35.0, 48.4)	57.8 (46.9, 68.1)	33.3 (24.3, 43.8)	0.002	50.0 (32.0, 68.0)	33.8 (23.6, 45.7)	0.14
School staff member	9.4 (6.3, 13.7)	15.7 (9.3, 25.2)	17.8 (11.1, 27.2)	0.71	10.7 (3.4, 28.9)	1.4 (0.2, 9.6)	0.04
Stranger	10.3 (6.9, 15.2)	8.4 (4.0, 16.7)	7.8 (3.7, 15.5)	0.88	0	18.3 (10.8, 29.3)	0.004
Neighbor	3.9 (2.1, 7.0)	3.6 (1.2, 10.7)	7.8 (3.7, 15.5)	0.24	0	4.2 (1.3, 12.5)	0.19
Someone else*	6.7 (4.1, 10.8)	7.2 (3.3, 15.3)	8.9 (4.5, 16.9)	0.69	3.6 (0.5, 22.0)	7.0 (2.9, 16.0)	0.52

All figures are weighted percentages (95% CI); %s are column percentages and may not add up to 100, since participants could select more than one form of violence. p values are from F tests.

*Includes violence from the following: employer, health care provider, military/police, religious leader, and someone else known.

In examining the prevalence of past-year violence types by youths’ self-reported mode of HIV acquisition, we found no significant differences between groups (S2 Table).

In the sensitivity analysis, when restricting to two or more acts of psychological abuse from at least one perpetrator type, the prevalence of past-year psychological victimization decreased from 65.3% to 52.0% (S3 Table). The prevalence of any past-year victimization decreased from 73.5% to 64.1% and of past-year polyvictimization from 37.8% to 34.1%.

### Perpetrator types of past-year violence

**Fig 3:**
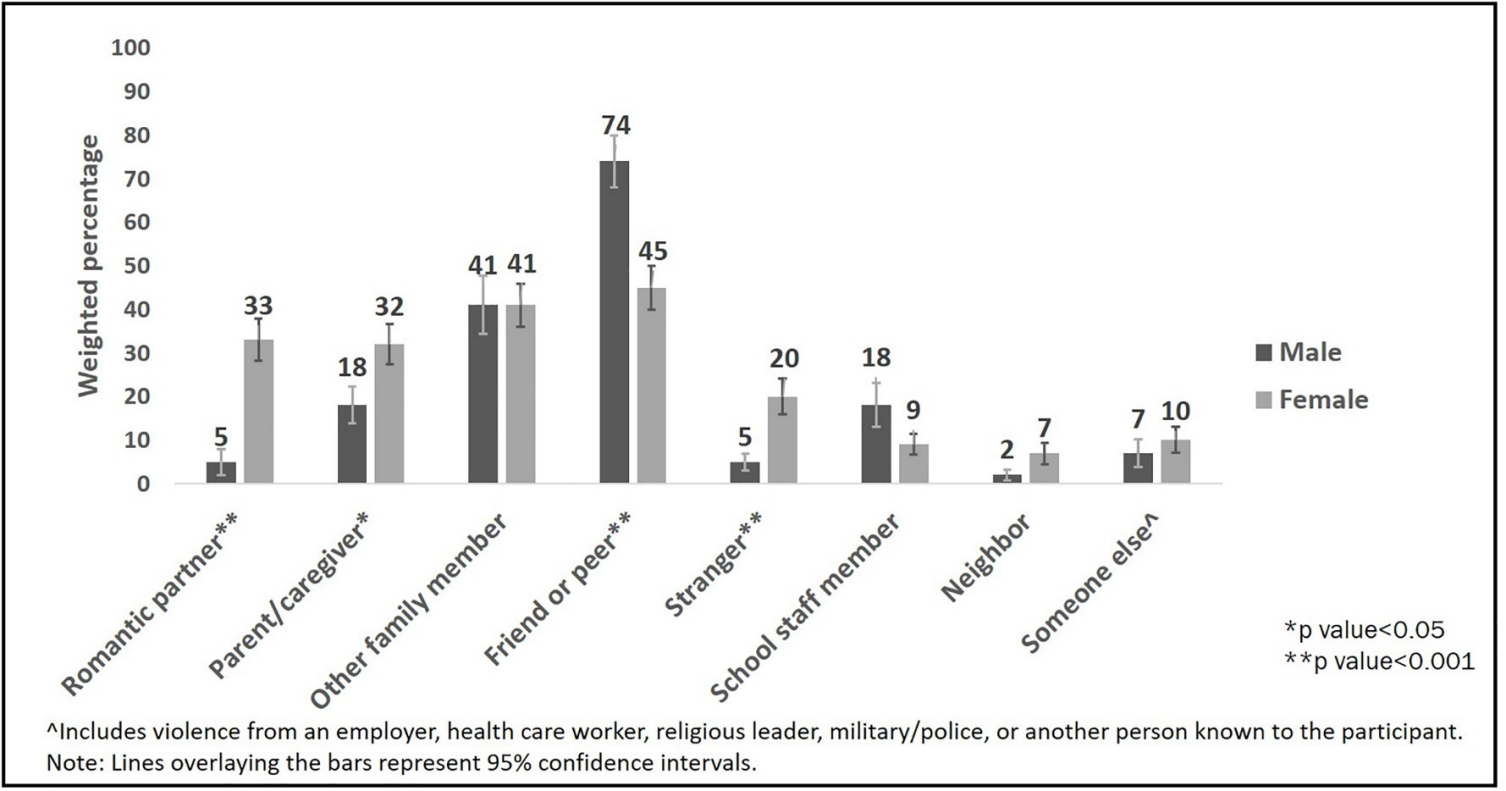
Differences in the perpetrators of any past-year violence (physical violence, psychological abuse, or forced sex) against HIV-positive adolescent and young adult victims of past-year violence, according to the youth’s sex.

Among past-year victims of violence, females compared to males experienced significantly higher levels of any past-year violence from a romantic partner (33.3% vs. 5.0%, p<0.001), parent/caregiver (32.4% vs. 17.6%, p = 0.02), and stranger (19.7% vs. 5.2%, p<0.001) (Fig 3 and Table 5). Both male and female victims of past-year violence experienced high levels from a friend or peer, especially psychological abuse. However, compared to females, male victims of past-year violence had significantly higher levels of any friend/peer victimization (74.3% vs. 45.1%, p<0.001), with the strongest evidence for a difference by sex found for physical violence (compared to psychological abuse) from a friend/peer. Experiencing violence from multiple perpetrator types was common, as less than half of past-year victims were victims of violence from a single perpetrator type (42.0%).

**Table 5:** Perpetrators of past-year violence against adolescents and young adults living with HIV in Zambia, among those who report past-year violence, stratified by sex.

	Total	Male	Female	
	% (95%CI)	% (95%CI)	% (95%CI)	p value^
Any victimization (n = 199)				
Romantic partner	22.1 (16.2, 29.6)	5.0 (1.5, 15.4)	33.3 (24.5, 43.4)	<0.001
Parent/ caregiver	26.6 (20.6, 33.7)	17.6 (10.7, 27.5)	32.4 (24.0, 42.1)	0.02
Other family member	41.1 (33.6, 49.1)	40.7 (28.5, 54.1)	41.4 (32.1, 51.4)	0.93
Friend or peer	56.5 (48.5, 64.2)	74.3 (61.1, 84.2)	45.1 (35.6, 55.1)	<0.001
School staff member	12.7 (8.6, 18.4)	17.9 (10.0, 30.0)	9.4 (5.7, 15.2)	0.09
Stranger	14.1 (9.4, 20.4)	5.2 (2.4, 10.8)	19.7 (12.8, 29.2)	<0.001
Neighbor	5.3 (2.9, 9.5)	2.2 (0.7, 6.8)	7.3 (3.7, 13.9)	0.06
Someone else**	9.1 (5.6, 14.5)	7.2 (2.9, 16.7)	10.3 (5.7, 17.8)	0.50
Physical violence (n = 121)				
Romantic partner	17.0 (10.5, 26.4)	0	27.7 (17.5, 40.8)	<0.001
Parent/ caregiver	28.3 (20.6, 37.5)	17.3 (9.7, 28.8)	35.3 (24.5, 47.7)	0.02
Other family member	27.2 (19.5, 36.6)	22.8 (11.9, 39.1)	30.0 (20.2, 42.0)	0.43
Friend or peer	34.3 (25.3, 44.5)	59.4 (42.4, 74.4)	18.5 (10.8, 29.8)	<0.001
School staff member	20.9 (14.3, 29.7)	29.9 (17.0, 47.0)	15.4 (9.3, 24.4)	0.07
Stranger	2.3 (0.6, 7.7)	0	3.7 (1.1, 12.2)	0.21
Neighbor	0.5 (0.0, 3.4)	1.2 (0.2, 8.6)	0	0.22
Someone else**	2.7 (0.9, 7.8)	3.7 (1.1, 11.3)	2.0 (0.3, 13.3)	0.60
Psychological abuse (n = 177)				
Romantic partner	16.3 (10.8, 23.8)	3.9 (0.8, 17.2)	24.5 (16.3, 35.1)	0.01
Parent/ caregiver	19.5 (13.8, 26.6)	11.2 (5.5, 21.4)	24.9 (16.9, 35.0)	0.03
Other family member	37.9 (30.1, 46.5)	40.7 (27.9, 55.0)	36.1 (26.6, 46.7)	0.59
Friend or peer	53.7 (45.2, 62.0)	67.8 (53.5, 79.4)	44.3 (34.1, 55.1)	0.01
School staff member	0.4 (0, 2.5)	0	0.6 (0, 4.1)	0.42
Stranger	14.6 (9.7, 21.5)	5.7 (2.6, 11.9)	20.5 (13.0, 30.9)	<0.01
Neighbor	5.7 (3.0, 10.4)	1.6 (0.4, 6.5)	8.3 (4.2, 15.8)	0.02
Someone else**	7.7 (4.4, 13.1)	1.6 (0.4, 6.5)	11.8 (6.5, 20.2)	0.00
Forced sex (n = 13)				
Romantic partner	57.2 (24.3, 84.8)	9.1 (1.7, 36.4)	48.2 (19.1, 78.5)	0.13
Parent/ caregiver	0	0	0	//
Other family member	0	0	0	//
Friend or peer	9.4 (1.8, 37.4)	4.5 (0.5, 33.3)	4.9 (0.5, 35.0)	0.72
School staff member	0	0	0	//
Stranger	0	0	0	//
Neighbor	0	0	0	//
Someone else**	33.4 (9.1, 71.4)	21.5 (3.7, 66.2)	11.9 (1.3, 58.7)	0.20
Number of perpetrator types* (n = 201)				
1 perpetrator type	42.0 (34.3, 50.2)	46.8 (33.9, 60.1)	39.0 (29.8, 49.1)	0.09
2 perpetrator types	34.2 (27.1, 42.1)	39.4 (27.4, 52.7)	30.8 (22.5, 40.7)	
3 perpetrator types	18.2 (13.1, 24.7)	10.9 (5.6, 20.3)	22.9 (15.7, 32.0)	
4+ perpetrator types	5.6 (3.1, 9.8)	3.0 (1.1, 7.8)	7.3 (3.7, 13.9)	

All n's and percentages are weighted; %s are column percentages and may not add up to 100, since participants could select more than one form of violence; percentages are for perpetrator types among those reporting a given form of violence; p values are from F tests.

*Of those reporting any violence victimization

**Includes violence from the following: employer, health care provider, military/police, religious leader, and someone else known to the youth.

## Discussion

Almost three-quarters of HIV-positive AYA, aged 15–24 years, in Zambia are estimated to have experienced both lifetime and past-year violence victimization, which is especially concerning since violence victimization may relate to poor HIV-care and treatment outcomes among HIV-positive youth in SSA [22]. Similar to several Violence against Children Surveys (VACS) among adolescents in the region [37, 42–44], we found no statistically significant differences in the overall prevalence of physical violence and psychological abuse according to the youth’s sex. However, we saw weak evidence that the prevalence of past-year *severe* physical violence was almost twice as high among females than males. We also identified nuanced differences in the types of perpetrators: friends and peers were a dominant perpetrator of physical and psychological abuse against males, whereas experiences of violence for females spanned more evenly across a range of perpetrators (i.e. romantic partners, parents/caregivers, other family members, friends/peers). These findings are important because previous studies of violence against HIV-positive youth in SSA, albeit limited in number, have neither distinguished between severity levels of physical violence experienced nor disaggregated perpetrators of violence according to the youth’s sex [20, 22].

Findings from this study have clinical and programmatic implications. HIV clinics offer a prime setting for identifying and supporting AYA living with HIV who have experienced violence. These clinics must recognize that a large proportion of their AYA patient populations may have experienced violence and need psychosocial support. While some SSA settings screen women for IPV in HIV centers as a standard care procedure [45], HIV-positive AYA should also be screened for multiple types of violence. AYA could be asked whether a given form of violence has occurred and who perpetrated the violence, with the perpetrator types read aloud to help prevent under-reporting. The Abuse Assessment Screening tool, designed for clinical screening of abuse during pregnancy [46], adopts a similar approach and could be adapted for such purposes. To make a screening program successful, however, additional resources either in the clinic or in the community are needed to fulfill the long-term support needs of AYA who have experienced and may continue to experience violence. Beyond clinics, our findings reinforce the need for broader violence-reduction efforts targeting general populations of AYA. We found that violence from friends or peers and family members was common, supporting violence prevention initiatives targeting schools and households [47, 48]. Our results suggest that for HIV-positive AYA, household interventions must engage other family members in the home beyond parents/caregivers. Finally, prevalence differences for male and female AYA highlight the need to tailor prevention and response efforts to the youth’s sex and the perpetrator type. For instance, programs designed to meet the unique needs of adolescent girls and young women living with HIV can integrate violence prevention and response content with a focus on the most common forms and perpetrators.

Our findings also have implications for the measurement of psychological abuse and sexual violence. Results from our sensitivity analysis reinforce calls in the literature to expand data collection efforts and clarify operational definitions for psychological abuse [38, 39]. Compared to physical and sexual violence, psychological abuse has received considerably less attention in studies of violence and HIV, reflecting its challenging measurement properties and a perception among some that it does less harm than physical or sexual violence [38, 49]. A recent publication of WHO MCS data seeking clarity on measurement of psychological IPV will support these efforts [50]. Importantly, our results underscore the need for such initiatives to explore the measurement of psychological abuse among male as well as female HIV-positive AYA and to include multiple perpetrator types.

Our removal of three ICAST-C measures of sexual violence suggests value in further testing these items among older youth in intimate partnerships. Youth may not interpret being “made” to do something as an act of coercion [51], particularly where items have been translated into other languages. The results regarding forced sex, however, found that male and female HIV-positive AYA have similar prevalence estimates. Although surprising since reports are typically higher among females compared with male adolescents in SSA [42, 43]—including in Zambia [37]—one study conducted with adolescents in South Africa (aged 14–19 years) also found no significant differences in the lifetime prevalence of forced sex victimization (14.3% among females and 10.8% among males) [52]. Taken together, these findings suggest a need for further research on the measurement and experience of sexual violence among young HIV-positive men in addition to women in the region.

While methodological variations between studies make direct comparisons difficult, we situated our findings within existing literature by comparing them with studies that have also examined experiences of multiple forms of violence from a wide range of perpetrators. Our prevalence figures for past-year violence from caregivers and peers were similar to those reported by Cluver et al. in their study among HIV-positive adolescents (ages 10–19 years) in South Africa [22]. However, more than twice as many youth reported violence from a teacher/school staff member in the Cluver study (41%) compared to in our study (18% of youth aged 15–19). Additionally, whereas about a fifth of youth in the Cluver study reported past-year verbal victimization from a clinician (22%), virtually no participants in our study reported violence from a health care worker (grouped within the “someone else” category in Tables 4 and 5). It may be that youth in our study felt less comfortable reporting experiences of violence from authority figures (e.g. teachers, clinicians) since surveys were being conducted within HIV clinics. Compared to the VACS among general populations of adolescents in Zambia [37] and other SSA countries [42, 43], we found a roughly similar prevalence of physical violence but higher levels of psychological abuse—even in sensitivity analyses using a more conservative measure of psychological abuse. These findings offer additional support for further research on experiences of psychological abuse among HIV-positive youth in SSA.

### Limitations and strengths

There are limits to this analysis. Given that AYA were sampled from purposively-selected clinics in an urban area and had been on ART for at least 6 months, our findings may not be generalizable to AYA who are living in rural areas or not accessing HIV care/on ART. Results may underestimate the prevalence of violence since those who have experienced violence tend to be less likely to engage in HIV care than those who do not [53, 54]. We did not ask youth if the violence they experienced was due to their HIV status—nor did we ask about, and therefore cannot discuss, the sexual orientation of the youth or the sex of the romantic partner perpetrating violence, though sexual minorities may be at higher risk of violence than heterosexuals [55]. Furthermore, we did not measure the perpetrator type for lifetime violence exposures or whether the lifetime physical violence or psychological abuse exposure occurred in early childhood versus adolescence, which limits the comprehensiveness of our lifetime victimization results. We may not have captured the full range of youth’s sexual violence experiences beyond physical force, in analyzing only one sexual violence measure. The lower-than-desired Cronbach’s alphas for physical violence and psychological abuse may indicate that these measures did not perform as well as in the populations in which they were developed or were diluted by the presence of sub-categories of violence. Since Cronbach’s alphas are influenced by a number of factors (e.g. test length, dimensionality) [36], future studies should include a formal assessment of the reliability of these measures among HIV-positive youth. Finally, the precision of our comparisons of victimization experiences by sex and age group may be limited by the small stratified sample sizes, though we note that this study is descriptive in scope and intended to generate rather than test hypotheses.

Despite these limitations, this study fills an important gap in the literature on violence against HIV-positive AYA in SSA. Key strengths of this study include its focus on multiple types and perpetrators of violence against HIV-positive AYA, and its inclusion of both males and females, as well as adolescents (15–19 years) and young adults (20–24 years). In contrast, large international surveys have typically been limited to examining experiences of violence among general populations of either children up to age 18 (e.g. CDC’s VACS) or women only (e.g. DHS). There has been virtually no previous research on violence victimization among young HIV-positive men ages 20–24 years in SSA.

## Conclusions

Our findings from Zambia demonstrate that experiences of violence victimization are widespread among youth living with HIV, highlighting the need for greater attention to this public health problem among HIV-positive youth in the region. Study findings show the importance of developing targeted prevention and response efforts that are tailored to the youth’s sex and the perpetrator type. Such efforts are critical to addressing the harmful short- and long-term effects of violence and may also help to prevent the onward transmission of HIV. Future studies should further investigate the intersections between violence victimization and HIV care and treatment outcomes, as well as meanings and operational definitions of psychological abuse and sexual violence, among HIV-positive AYA in sub-Saharan Africa.

## Supporting information

S1 Table Lifetime and past-year prevalence of individual acts of violence victimization among adolescents and young adults living with HIV in Zambia, stratified by sex. (DOCX)

S2 Table Estimated past-year prevalence of violence victimization among adolescents and young adults living with HIV in Ndola, Zambia, stratified by self-reported mode of HIV acquisition (n = 272). (DOCX)

S3 Table Past-year prevalence of violence victimization among adolescents and young adults living with HIV in Zambia, stratified by sex, restricting to 2+ acts of psychological abuse from at least one perpetrator type (n = 272). (DOCX)

S1 Data Soar transition study—Baseline survey. (DOCX)
