# Supplementary material for: Prevalence of physical and sexual violence and psychological abuse among adolescents and young adults living with HIV in Zambia
Source: PLoS One. 2020 Jun 25;15(6):e0235203. doi: 10.1371/journal.pone.0235203 (PMC7316234; doi:10.1371/journal.pone.0235203)
Supplement: S1 Table — (DOCX) [file pone.0235203.s001.docx]

|  | **Lifetime** | | **Past-year** | | **Past-year prevalence, by sex** | | | | |
| --- | --- | --- | --- | --- | --- | --- | --- | --- | --- |
|  |  |  |  |  | **Male** | | **Female** | | **p value** |
| **Physical violence** |  |  |  |  |  |  |  |  |  |
| Slapped or thrown at by something | 32.4 | (26.5, 39.0) | 28.4 | (22.8, 34.8) | 23.3 | (15.4, 33.7) | 31.8 | (24.2, 39.8) | 0.18 |
| Pushed or shoved | 15.5 | (11.2, 20.9) | 14.4 | (10.2, 19.6) | 16.5 | (9.8, 26.4) | 13.0 | (8.3, 19.6) | 0.49 |
| Ear or arm twisted as punishment | 24.3 | (19.0, 30.4) | 20.8 | (15.9, 26.7) | 22.2 | (14.4, 32.6) | 19.9 | (14.1, 27.3) | 0.68 |
| Hit with a fist or with something else that could hurt (e.g. stick/cane) | 15.2 | (11.0, 20.5) | 12.6 | (8.9, 17.6) | 8.8 | (4.3, 17.2) | 15.2 | (10.1, 22.0) | 0.17 |
| Kicked, dragged, or severely beaten up | 4.8 | (2.7, 8.4) | 4.6 | (2.5, 8.2) | 1.6 | (0.5, 4.9) | 6.5 | (3.3, 12.2) | 0.02 |
| Choked or burnt on purpose | 0.9 | (0.3, 2.4) | 0.9 | (0.3 2.4) | 0.5 | (0, 3.8) | 1.1 | (3.6, 3.5) | 0.50 |
| Threatened with/used a sharp object or other weapon | 3.7 | (1.9, 7.2) | 2.3 | (1.2, 4.7) | 1.1 | (0.3, 4.3) | 3.2 | (1.4, 6.9) | 0.16 |
| **Psychological abuse** |  |  |  |  |  |  |  |  |  |
| Insulted or made to feel bad | 64.9 | (58.2, 71.0) | 59.7 | (52.9, 66.2) | 58.1 | (46.5, 68.9) | 60.8 | (52.4, 68.6) | 0.70 |
| Belittled or humiliated in front of other people | 31.7 | (25.7, 38.3) | 29.5 | (23.6, 36.1) | 27.8 | (18.9, 38.9) | 30.6 | (23.3, 39.0) | 0.67 |
| Threatened with leave or abandonment | 18.8 | (14.1, 24.7) | 17.2 | (12.7, 22.8) | 13.4 | (7.6, 22.7) | 19.8 | (13.9, 27.4) | 0.23 |
| Locked inside or outside the home | 9.8 | (6.5, 14.6) | 8.1 | (5.1, 12.5) | 1.1 | (0.3, 4.2) | 12.7 | (7.9, 19.7) | <0.001 |
| Threatened with harmful people, ghosts or evil spirits | 5.3 | (3.0, 9.1) | 3.8 | (2.0, 7.4) | 3.6 | (1.1, 11.3) | 4.1 | (1.9, 8.6) | 0.84 |
| Skin color/ gender/ religion/ tribe/ or health problems referred to in hurtful way | 16.7 | (12.1, 22.5) | 13.3 | (9.3, 18.7) | 12.8 | (6.7, 22.9) | 13.7 | (8.9, 20.5) | 0.86 |
| **Forced sex** |  |  |  |  |  |  |  |  |  |
| Physically forced to have sexual intercourse when did not want to | 10.4 | (6.9, 15.5) | 4.7 | (2.6, 8.3) | 4.1 | (1.4, 11.5) | 5.0 | (2.4, 10.0) | 0.75 |
| Notes: Figures are weighted proportion (95% confidence interval); %s are column percentages and may not add up to 100, since participants could select more than one form of violence. p values are from F tests. | | | | | | | | | |

**S1 Table: Lifetime and past-year prevalence of individual acts of violence victimization among adolescents and young adults living with HIV in Zambia, stratified by sex**
