# Supplementary material for: Prevalence of physical and sexual violence and psychological abuse among adolescents and young adults living with HIV in Zambia
Source: PLoS One. 2020 Jun 25;15(6):e0235203. doi: 10.1371/journal.pone.0235203 (PMC7316234; doi:10.1371/journal.pone.0235203)
Supplement: S2 Table — (DOCX) [file pone.0235203.s002.docx]

**S2 Table: Estimated past-year prevalence of violence victimization among adolescents and young adults living with HIV in Ndola, Zambia, stratified by self-reported mode of HIV acquisition (n=272)**

|  | **HIV acquisition from parents (n=193) % (95%CI)** | **HIV acquisition through other means^ (n=79) % (95%CI)** | **p value** |
| --- | --- | --- | --- |
| **Any victimization** |  |  |  |
| Physical, psychological, or forced sex | 75.2 (67.5, 81.6) | 69.3 (56.4, 79.7) | 0.38 |
| **Type of victimization** |  |  |  |
| Physical violence^^ | 47.6 (39.7, 55.7) | 37.5 (26.3, 50.2) | 0.18 |
| Moderate physical violence | 44.8 (37.0, 52.9) | 37.5 (26.3, 50.2) | 0.33 |
| Severe physical violence | 15.1 (10.4, 21.4) | 15.4 (8.3, 26.8) | 0.95 |
| Psychological abuse | 66.8 (58.8, 74.0) | 61.6 (48.8, 73.0) | 0.47 |
| Forced sex | 5.2 (2.6, 10.0) | 3.4 (1.0, 11.4) | 0.56 |
| **Polyvictimization*** |  |  |  |
| No violence | 24.8 (18.4, 32.5) | 30.7 (20.3, 43.6) | 0.32 |
| 1 type of violence | 34.2 (27.0, 42.4) | 39.4 (27.8, 52.3) |  |
| 2+ types of violence | 41.0 (33.3, 49.1) | 29.9 (19.7, 42.5) |  |
| Notes: n’s and percentages are weighted; %s are column percentages and may not add up to 100, since participants could select more than one form of violence. p values are from F tests. ^Response options included “through sex,” “another way,” and “don’t know/refused.”  ^^Percentages for moderate and severe physical violence are among those reporting any physical violence (n=212 lifetime, n=199 past-year). *Categories are mutually exclusive. | | | |
