# Supplementary material for: Prevalence of physical and sexual violence and psychological abuse among adolescents and young adults living with HIV in Zambia
Source: PLoS One. 2020 Jun 25;15(6):e0235203. doi: 10.1371/journal.pone.0235203 (PMC7316234; doi:10.1371/journal.pone.0235203)
Supplement: S3 Table — (DOCX) [file pone.0235203.s003.docx]

| **S3 Table: Past-year prevalence of violence victimization among adolescents and young adults living with HIV in Zambia, stratified by sex, restricting to 2+ acts of psychological abuse from at least one perpetrator group (n=272)** | | | | | | | |
| --- | --- | --- | --- | --- | --- | --- | --- |
| **Violence victimization** | **Total** | | **Stratified by sex** | | | | |
|  |  |  | **Male** | | **Female** | |  |
|  | **%** | **(95%CI)** | **%** | **(95%CI)** | **%** | **(95%CI)** | **p value^** |
| Any violence | 64.1 | (57.3, 70.4) | 65.2 | (53.6, 75.2) | 63.4 | (54.9, 71.1) | 0.80 |
| Psychological abuse only | 52.0 | (45.2, 58.8) | 56.9 | (45.4, 67.7) | 48.7 | (40.4, 57.2) | 0.26 |
| Polyvictimization |  |  |  |  |  |  |  |
| No violence | 35.9 | (30.0, 42.7) | 34.8 | (24.8, 46.4) | 36.6 | (28.9, 45.1) | 0.77 |
| 1 type of violence | 30.0 | (24.1, 36.6) | 28.2 | (19.1, 39.5) | 31.2 | (23.9, 39.6) |  |
| 2+ types of violence | 34.1 | (27.9, 40.8) | 37.0 | (26.8, 48.5) | 32.2 | (24.8, 40.5) |  |
| Notes: Percentages are weighted; %s are column percentages and may not add up to 100, since participants could select more than one form of violence. p values are from F tests. Respondents classified as non-victims if reporting a single act of psychological abuse from a single perpetrator group. | | | | | | | |
