# Supplementary material for: Prevalence of physical and sexual violence and psychological abuse among adolescents and young adults living with HIV in Zambia
Source: PLoS One. 2020 Jun 25;15(6):e0235203. doi: 10.1371/journal.pone.0235203 (PMC7316234; doi:10.1371/journal.pone.0235203)
Supplement: S1 Data — (DOCX) [file pone.0235203.s004.docx]

| **SOAR TRANSITION STUDY – BASELINE SURVEY**  **December 21, 2017 V 3.0** | | | |
| --- | --- | --- | --- |
| **Q#** | **Question** | **Answers** | **Special Instructions** |
| **Introduction** | | | |
| Thank you for agreeing to participate in the study. I am now going to start asking you questions and recording you responses.  Natotela pakusangwamo muli uku ku fwaikisha. Nalamipushako amepusho kabili nkalalemba efyomukala asuka. | | | |
| **Interview Information** | | | |
|  | Date of Interview |  |  |
| **Patient Identifiers** - Study staff fill out | | | |
|  | Record participant's study ID Number |  |  |
|  | Study Facility | 01 ADCH 02 NTH 03 Lubuto Clinic 04 Twapia Clinic |  |
| **Socio-Demographics** | | | |
|  | Male or Female?  Bushe mwaume /mwanakashi | 01 Male  02 Female  888 Refused to answer  01 Mwaume 02 Mwana Kashi  888 Akana kwasuka |  |
|  | How old are you now?  Bushe uli ne myaka iinga pali lelo? | 15 16 17 18 19 20 21 22 23 24  888 Akana kwasuka |  |
|  | Have you ever attended school?  Bushe walipitaako ku sukulu? | 00 No 01 Yes 777 Don't know 888 Refused to answer  00 Awe 01 Emukwai 777 Teshibe 888 Akana ukwasuka | *If NO, skip to Q12.*  *If YES, proceed to next question.* |
|  | Have you completed primary school?  Bushe walipwisha amasambililo yaku pulalimali? | 00 No 01 Yes 777 Don't know 888 Refused to answer  00 Awe 01 Emukwai 777 Teshibe 888 Akana ukwasuka | *If NO, skip to Q9.*  *If YES, proceed to next question.* |
|  | Have you completed secondary school?  Bushe walipwisha amasambililo yaku sekondali? | 00 No 01 Yes 888 Refused to answer  00 Awe 01 Emukwai 888 Akana ukwasuka |  |
|  | Are you currently in school?  Bushe ucilli pa sukulu? | 00 No 01 Yes 888 Refused to answer  00 Awe 01 Emukwai 888 Akana ukwasuka | *If NO, skip to Q12*  *If YES, proceed to next question.* |
|  | What school grade are you currently in?  Bushe pali ino nshita uli mu grade shani? | 01-12 13 Enrolled in University, College, or Technical School  888 Refused to answer  01-12  13- Nalilembesha ku University nangu ku Tekeniko  888 Akana ukwasuka |  |
|  | What type of school do you go to?  Bushe li sukulu lyashani uyako? | 01 Day school 02 Boarding school 03 University/College/Technical School 04 Other, specify _____.  888 Refused to answer  01 isukulu yakuya nokuwela  02 Isukulu yakusendama kulya kwine  03 Ku University/ Tekeniko  04 Kumbifye, landa  888 Akana kwasuka | *If participant selects "Boarding school", prompt appears: “Participant has reported that he or she is attending a boarding school. This is an exclusion criteria. Please discontinue survey.”* |
|  | Other (specify) |  |  |
|  | Are you currently employed?  Bushe pali ino nshita ula bomba? | 00 No  01 Yes  888 Refused to answer  00 Awe 01 Emukwai  888 Akana ukwasuka |  |
|  | How do you think you acquired HIV?  Bushe utontonkanya ati nimumusango nshi wa ambwilemo aka shishi ka HIV? | 01 From your parents 02 Through sex 03 Another way 777 Don’t know 888 Refused to answer  01 Ukufuma kuba fyashi  02 Ukupitila mukupanga icupo  03 Munshila imbi  777 Teshibe  888 Akana Kwasuka |  |
| **Family Characteristics** | | | |
|  | Is your biological mother alive?  Bushe abafyashi abanakashi aba kufyala epo baba? | 00 No 01 Yes  777 Don’t Know 888 Refused to answer  00 Awe 01 Emukwai  777Teshibe 888 Akana ukwasuka |  |
|  | Is your biological father alive?  Bushe abafyashi abaume aba kufyala epo baba? | 00 No 01 Yes 777 Don’t know 888 Refused to answer  00 Awe 01 Emukwai  777 Teshibe 888 Akana ukwasuka |  |
|  | Who do you typically live with?  Nibanani wikala nabo pali inonshita? | *Select all that apply:*  01 Biological Mom 02 Biological Dad 03 Other family 04 Orphanage 05 girlfriend/boyfriend  06 Wife/Husband  07 Other  888 Refused to answer  *Saleni pali ifyo fyonse ifikumineko*  01 Banyina aba mufyala  02 Bawishi aba mufyala  03 Bambi mulupwa  04 Ku Ophanage  05 girlfriend/boyfriend  06 Umukashi/umulume  07 Bambi  888 Akana ukwasuka |  |
|  | Are there other people in your home living with HIV?  Bushe mu ng’anda wikala, mwalibako nabambi abakwata aka shishi ka HIV? | 00 No 01 Yes 777 Don't Know 888 Refused to answer  00 Awe 01 Emukwai  777 Teshibe 888 Akana ukwasuka | *If NO/DK/Refused, skip to Q19.*  *If YES, proceed to Q18.* |
|  | If Yes, are any of them on ART?  Nga epo baba, bushe paliba abanwa umuti uwama ARV? | 00 No 01 Yes 777 Don't Know 888 Refused to answer  00 Awe 01Emukwai  777 Teshibe  888 Akana ukwasuka |  |
|  | How many families/households have you lived in?  Bushe nindupwa shinga/ nangula mayanda yanga waikalamo? | 01 02 03 04 05 06 07 08 09 10 11 More than 10 / Ukucila pe kumi 777 sse / Teshibe 888 Refused to Answer / Akana ukwasuka |  |
|  | What is your marital status?  Bushe walyupwa / walyupa? | 01 Single (never married) 02 Married  03 Separated/divorced  04 Widowed  05 Living together  888 Refused to answer  01 Mushimbe (Taupwapo/Taupapo) 02 Nalyupwa/nalyupa  03 Bapusana/icupo calipwa  04 Muka nfwilwa  05 Ukwikala pamo  888 Akana ukwasuka |  |
| **Family Engagement/Belonging** | | | |
|  | *In the past 4 weeks…* *Mumilungu 4 yapita….*  you felt you had a strong relationship with your family.  walyunfwile ukukwata isenge sana naba lupwa Bobe. | 00 Never 01 Rarely 02 Sometimes 03 Often 04 Always  888 Refused to answer  00 Awe nakalya 01 Patali patali 02 Limolimo 03 lyonsefye 04 Sanafye  888 Akana ukwasuka | [read response options] |
|  | *In the past 4 weeks… Mumilungu 4 yapita….*  you felt really important to your family.  walyunfwile ukuba uwacidama kuli ba lupwa bobe. | 00 Never 01 Rarely 02 Sometimes 03 Often 04 Always  888 Refused to answer  00 Awe nakalya 01 Patali patali 02 Limolimo 03 lyonsefye 04 Sanafye  888 Akana ukwasuka | [read response options] |
|  | *In the past 4 weeks…* *Mumilungu 4 yapita….*  you got all the help you needed from your family.  walipokele bonse ubwafwilisho walekabila ukufuma kuli ba lupwa .bobe. | 00 Never 01 Rarely 02 Sometimes 03 Often 04 Always  888 Refused to answer  00 Awe nakalya 01 Patali patali 02 Limolimo 03 lyonsefye 04 Sanafye  888 Akana ukwasuka | [read response options] |
|  | *In the past 4 weeks…* *Mumilungu 4 yapita….*  your family and you had fun together.  iwe pamo nabalupwa bobe mwalisansamukila capamo | 00 Never 01 Rarely 02 Sometimes 03 Often 04 Always  888 Refused to answer  00 Awe nakalya 01 Patali patali 02 Limolimo 03 lyonsefye 04 Sanafye  888 Akana ukwasuka | [read response options] |
|  | *In the past 4 weeks…* *Mumilungu 4 yapita….*  People in your family made you feel good about yourself  abantu mu mulumpwa lobe balengele iwe ukunfwa bwino | 00 Never 01 Rarely 02 Sometimes 03 Often 04 Always  888 Refused to answer  00 Awe nakalya 01 Patali patali 02 Limolimo 03 lyonsefye 04 Sanafye  888 Akana ukwasuka | [read response options] |
|  | *In the past 4 weeks…* *Mumilungu 4 yapita….*  your family treated you fairly.  Balupwa bobe balikusungile bwino | 00 Never 01 Rarely 02 Sometimes 03 Often 04 Always  888 Refused to answer  00 Awe nakalya 01 Patali patali 02 Limolimo 03 lyonsefye 04 Sanafye  888 Akana ukwasuka | [read response options] |
|  | *In the past 4 weeks…* *Mumilungu 4 yapita….*  your parents listened to you .  Aba fyashi bobe balakumfwa nga walanda | 00 Never 01 Rarely 02 Sometimes 03 Often 04 Always  888 Refused to answer  00 Awe nakalya 01 Patali patali 02 Limolimo 03 lyonsefye 04 Sanafye  888 Akana ukwasuka | [read response options] |
|  | *In the past 4 weeks…* *Mumilungu 4 yapita….*  your family paid a lot of attention to you  Balupwa bobe balikuposako amano. | 00 Never 01 Rarely 02 Sometimes 03 Often 04 Always  888 Refused to answer  00 Awe nakalya 01 Patali patali 02 Limolimo 03 lyonsefye 04 Sanafye  888 Akana kwasuka | [read response options] |
| **Disclosing to Others / Ukwebako abantu bambi** | | | |
| Survey administrator reads: *Now we would like to ask you some questions about your life living with HIV. These questions may make you feel anxious or sad. You do not have to answer any questions you do not want to. Please remember that your answers will be kept confidential.*  Mukalamba wapali uku kufwailikisha atampako kubelenga: Nomba twa lafwayako uku mipushako yamo amepusho pa bwikashi bwenu na HIV. Aya amepusho kuti yalenga kumyunfwisha umweso nangula ifibi. Tamulingile ukwasuka efyo tamulefwaya ukwasuka. Mukwai mwishibe ati ifyo mwalatweba fika sungwa munkama. | | | |
|  | Would you say that no one, a few people, most people, or everyone in the home you currently live in know that you are living with HIV?  Bushe kuti watila takwaba uwaishiba, banono fye baishiba, abengi nangu bonse abo wikala nabo pa ng’anda balishiba ati walikwata HIV? | 00 No one 01 A few people 02 Most people 03 Everyone 777 Don't Know 888 Refused to answer 999 Not applicable - live alone  00 Tapaba nangu umo  01 Ba nonofye baishiba  02 Abengi balishiba  03 Bonse  777 Teshibe  888 Akana ukwasuka  999 Tafimukumine-ekala eka | *If Everyone or NA, skip to Q31.* |
|  | If not everyone knows, who is not aware of your HIV status?  Nga tebonse abaishiba, ati walikwata akashishi ka HIV bushe ninshi nibani bashaishiba? | 01 adults 02 children 03 both adults and children 777 Don't know 888 Refused to answer  999 Not applicable – everyone knows  01 Aba kalamba  02 Abaice  03 Bonse aba kalamba na baice  777 Teshibe  888 Akana ukwasuka  999 Tafimukumine- bonse balishiba |  |
| **Learning your HIV status / Ukusambilila ubumi bobe ubwa HIV** | | | |
|  | How old were you when you learned you were living with HIV?  Wali ne myaka inga ilyo waishibe ukuti walikwata HIV? | [__ __] years  (0-24)  *Record 777 for Don’t know or 888 for Refused to answer.*  [__ __] imyaka  (0-24)  *bikeni 777 nga tabeshibe imyaka nangu 888 nga tabalefwaya ukwasuka.* |  |
|  | Did someone tell you that you were living with HIV or did you figure it out on your own?  Bushe kwaliko aba kwebele ati wali kwata akashishi ka HIV nangula waishibilefye wemwine? | 01 Directly informed 02 Figured it out on your own 777 Don't Know  888 Refused to answer  01 Ba njebelefye  02 Nali shibilefye nemwine  777 Teshibe  888 Akana ukwasuka |  |
|  | Who was with you when you first learned you were living with HIV?  Nibani abali naiwe ilintu waishibe ati wali kwata akashishi ka HIV? | *Select all that apply:* 01 Health care provider 02 parent/caregiver 03 Other adult family member 04 Peer/Friend 05 Adult neighbor 06 Teacher  07 Religious leader  08 No-one 09 Other adult 777 Don’t Know 888 Refused to answer  Sala fyonse ifilingile:  01 Aba bonfi abalolekesha pafya bumi  02 Aba fyashi  03 Aba kalamba bambi mulupwa  04 Umu nandi  05 Aba kalamba Mucipanda  06 Ba kafundisha  07 Itungulushi yama Pepo  08 Takuli naumbi  09 Bambi aba kalamba  777 Teshibe  888 Akana ukwasuka | *Survey administrator should ask each answer option individually.* |
|  | Who was the first person to directly tell you that you were living with HIV?  Nibanani ba balilepo uku kweba ati walikwata aka shishi ka HIV? | *Select all that apply:*  01 Health care provider 02 parent/caregiver 03 Other adult family member 04 Peer/friend  05 Adult neighbor 06 Teacher 07 Religious leader 08 Other adult 777 Don’t Know 888 Refused to answer  Sala fyonse filingile:  01 Aba bonfi abalolekesha pafya bumi  02 Aba fyashi  03 Aba kalamba bambi mulupwa  04 Umu nandi  05 Abakalamba Mucipanda  06 Ba kafundisha 07 Itungulushi yama Pepo 08 Bambi aba kalamba  777 teshibe 888 akana ukwasuka | [read response options] |
|  | Where were you when you first learned you were living with HIV?  Nikwisa wali umuku wakubalilapo lintu waishibe ati walikwata aka shishi ka HIV? | 01 Health facility 02 Home 03 School 04 Community 05 Other 777 Don’t know 888 Refused to answer  01 Ku cipatala 02 Ku ng’anda 03 ku sukulu 04 Ku Cipanda 05 Kumbifye 777 Teshibe 888 Akana ukwasuka |  |
|  | Did you have people you could talk with about living with HIV after you found out your HIV status?  Bushe walikwete abantu aba kulanda nabo ilintu waishibe ati walikwata aka shishi ka HIV? | 00 No  01 Yes 888 Refused to answer  01 Awe 02 Emukwa 88 Akana ukwasuka |  |
|  | Do you wish your parent/caregiver had told you your HIV status earlier?  Bushe kuti wakabila ukuti nga bafyashi ba kwebele ati walikwata aka shishi ka HIV bwangu? | 00 No  01 Yes  888 Refused to answer  999 Not applicable  00 Awe  01 Emukwai  888 Akana ukwasuka  999 Tafilingile | *If NO, skip to Q39.* |
|  | At what age do you wish you had learned your HIV status?  Nipa myaka yinga ngelo watemenwe ukwishiba ati walikwata aka shishi ka HIV? | [0-25]  (for "Wish I had always known/known from birth", enter 0)  888 Refused to answer  888 Akana ukwasuka |  |
|  | Do you wish you had more time with a counselor at the health center when you learned your HIV status?  Bushe walekabila ukukwata inshita iyikulu naba counselor pa cipatala elyo waishibe ati walikwata aka shishi ka HIV? | 00 No  01 Yes  888 Refused to answer  999 Not applicable  00 Awe  01 Emukwai  88 Akana ukwasuka  99 Tafilingile |  |
|  | Do you wish you belonged to a peer support group immediately after learning your HIV status?  Bushe nga walitemenwe ukuikumika kuka bungwe kaba nobe akatungilila elyo waishibe ati naukwata aka shishi ka HIV? | 00 No, I don’t wish I’d had a group immediately.  01 Yes, I do wish I’d had a group immediately.  03 I did have a group immediately.  888 Refused to answer  999 Not applicable (e.g. very young when learned his/her status)  00 Awe nshilekabila ati nga nalikwete ibumba apopene.  01 Emukwai, ndekabila nga nalikwete ibumba apopene.  03 Nalikwete ibumba apopene.  888 Akana ukwasuka  999 Tafilingile (e.g ali munono elyo aishibe ubumi bwakwe) |  |
| **ART medication self-management / Ukui tangata wemwine pa muti wama ARV** | | | |
|  | Who usually picks up your ART drugs from the clinic?  Ni banani bakupokela umuti uwama ARV ku kiliniki? | 01 I pick them up myself 02 Family/household member you live with 03 Other relatives you do not live with 04 Health care provider 05 Neighbor 06 Orphanage caretaker 07 Other, specify  888 Refused to answer  01 Ndaya mu kuipokela 02 Abapa myesu abo twikala nabo  03 Balupwa bambi abo nshikala nabo pa ng’anda 04 Ababomba ku Cipatala 05 Abena mupalamano 06 Abalolekesha pabana ba nshiwa 07 Bambi ( balumbule)  888 Akana ukwasuka |  |
|  | Other, specify: |  |  |
|  | Who keeps your ART drugs?  Ni banani baku sungila umuti uwama ARV? | 01 Self 02 Teacher 03 Adult you live with 04 Adult you do not live with (non-teacher) 05 Orphanage caretaker 06 Other, specify ______.  888 Refused to answer  01 Ne mwine 02 Bakafundisha 03 Abakalamba abo twikala nabo pa ng’anda  04 Abakalamba bambi ebo nshikala nabo pa ng’anda ( ukufimishako ba kafundisha)  05 Abalolekesha pa bana banshiwa 06 Bambi (balumbule)  88 Akana ukwasuka |  |
|  | Other, specify: |  |  |
|  | On a typical day, does someone watch you swallow your ART drugs to make sure you take them?  Bushe kwaliba bamo abaposako amano pa kuti ulenwa lyonse umuti wa ma ARV? | 00 No 01 Yes 888 Refused to answer  00 Awe 01 Emukwai 888 Akana ukwasuka | *If NO, skip to Q45.* |
|  | If Yes, who [watches you swallow your ART drugs to make sure you take them]?  Nga eko baba abakumona wanwa umuti wama ARV ninshi nibanani? | *Select all that apply:* 01 People you live with  02 Relatives you do not live with 03 People at school 04 Health care workers 05 Other people you do not live with  888 Refused to answer  *Sala fyonse ifilefwaika* 01 Abo njikala nabo  02 Ba lupwa abo tawikala nabo 03 Aba pa sukulu 04 Aba bonfi abalolekesha pafya bumi 05 Bambi abantu ebo tawikala nabo  888 Akana ukwasuka | *[Read responses]* |
|  | Do you typically forget to take your ART drugs unless someone reminds you to take them?  Bushe ilingi ulalaba ukunwa umuti uwama ARV kanofye kwaba abaku kwibukisha ukunwa? | 00 No 01 Yes 888 Refused to answer  00 Awe 01 Emukwai 888 Akana ukwasuka | *If NO, skip to Q47.* |
|  | If yes, who [reminds you to take your ART drugs]?  Nga eko bali, nibanani abakwibukishako ukunwa umuti uwama ARV? | 01 Only people at home 02 Only people outside of the home  03 People at home & outside of the home 888 Refused to answer  01 Aba pa ng’andafye 02 Abashili ba pa ng’andafye  03 aba pa ng’anda nabashili ba pang’anda 888 Akana ukwasuka |  |
|  | In the past three months, have you attended any adolescent club meetings at this clinic?  Mumyeshi shitatu shapita walisako ku adolescent club pano pa cipatala? | 00 No 01 Yes 777 Don’t know 888 Refused to answer  00 Awe 01 Emukwai  777 Teshibe  888 Akana ukwasuka | *If No, Don’t know, or Refused to answer, skip to Q49.* |
|  | If yes, how many did you attend?  Nga walishileko, miku yinga waishileko? | 00 None 01 One meeting  02 Two meetings 03 Three meetings 777 Don’t know 888 Refused to answer  00 Tapali 01 Muku umo 02 Miku ibili 03 Miku itatu 777 Teshibe 888 Akana ukwasuka |  |
|  | For your past three visits to this clinic, has anyone accompanied you?  Pamiku itatu esho waisako kuno ku cipatala, bushe kwali abakushindikeko? | 00 No 01 Yes  888 Refused to answer  00 Awe 01 Emukwai  777 Teshibe  888 Akana ukwasuka | *If No, proceed to Q50.*  *If Yes, skip to Q51.* |
|  | If NO, at what age did you start coming to this clinic alone?  ***Accept estimations***  Nga takuli, ninshi wali ne myaka inga ilyo watendeke ukwisa kuno ku kiliniki weka? | estimate 0-24  777 Don’t know  888 Refused to answer  777 teshibe  888 akana ukwasuka | *Skip to Q52.* |
|  | If YES, who usually accompanies you?  Ngekobali, bushe nibanani abakushindika? | *Select all that apply:* 01 Biological Mother 02 Biological Father 03 Other adult caregiver 04 Peer/friend 05 Teacher  06 Religious leader  10 Other 888 Refused to answer  *sala yonse ayalefwaika:* 01 Abafyashi abaku fyala abana kashi 02 Abafyashi abaku fyala abaume 03 Bambifye abakulu 04 Umunandi  05 Ba kafundisha 06 Intungulushi yamapepo 10 Bambifye  888 Akana ukwasuka |  |
|  | If no one is able to pick up your drugs for you, how confident are you that you can get to the clinic and pick up your drugs yourself?  Nga cakweba ati takuli abaku kupokelako umuti, bushe wakosa shani ukuya ku cipatala ukuya ipokela umuti wemwine? | 00 Not at all confident 01 Somewhat confident 02 Very confident  888 Refused to answer  00 Nshakosa sana 01 Nalikosako panono 02 Nalikosa sana  888 Akana ukwasuka |  |
|  | If no one is able to go to the clinic with you when you have an appointment, how confident are you that you are able to get to the clinic by yourself?  Nga cakweba ati takuli uwaku kushindika ku Cipatala mukukumona, bushe walikosa kuti wayila weka? | 00 Not at all confident 01 Somewhat confident 02 Very confident  888 Refused to answer  00 Nshakosa sana 01 Nalikosako panono 02 Nalikosa sana  888 Akana ukwasuka |  |
|  | Would you feel confident going to the clinic by yourself when you feel unwell, even if you do not have an appointment?  Bushe kuti wakosa ukuya ku kiliniki nga walyunfwile uku lwala nangu takuli abalekucetekela ku cipatala? | 00 Not at all confident 01 Somewhat confident 02 Very confident  888 Refused to answer  00 nshakosa sana 01 nalikosako panono 02 nalikosa sana  888 akana ukwasuka |  |
|  | *ADCH:* How confident would you feel going to an adult HIV clinic at NTH?  Bushe kuti wakosa shani, ukulaya ku Cipatala ca bakalamba ku HIV kilinik pa NTH?  *NTH/Twapia/Lubuto*: How confident would you feel going to adult clinic hours at NTH/Twapia/Lubuto?  Bushe kuti wakosa shani, ukulaya ku Cipatala ca bakalamba panshinta imoyine nabo pa NTH/Twapia/Lubuto? | 00 Not at all confident 01 Somewhat confident 02 Very confident  888 Refused to answer  00 nshakosa sana 01 nalikosako panono 02 nalikosa sana  888 akana ukwasuka | [Read Responses] |
| **HIV Disclosure Self-efficacy / Ukusokolola pabumi bwa HIV palobe** | | | |
| Survey administrator reads: *Now* *we will ask you some questions about disclosing your HIV status to other people.*  *Nomba twalamipushako amepusho pakwebako abantu bambi paka shishi aka HIV mwakwata.* | | | |
|  | Have you ever told anyone that you are living with HIV?  Bushe walyebako abali bonse ati walikwata akashishi ka HIV? | 00 No 01 Yes 888 Refused to Answer  00 Awe 01 Emukwai  888 Akana ukwasuka |  |
|  | Do you agree or disagree that disclosing your HIV status is your decision?  Bushe ulesuminisha nagula iyo ati ukwebako bambi ati walikwata ka shishi aka HIV ninsambu shobe? | 00 Disagree 01 Agree 888 Refused to Answer  00 Nakana 01 Nasumina  888 Akana ukwasuka |  |
|  | Has an adult in your life forbidden you from telling anyone your HIV status in the past year?  Bushe kwalibako abakalamba mumweo obe abakukanya ukwebako bambi ati walikwata aka shishi ka HIV mu myaka wapita? | 00 No  01 Yes 888 Refused to answer  00 Awe 01 Emukwai  888 Akana ukwasuka | *If YES, proceed to Q59*  *If NO, skip to Q60.* |
|  | If so, who?  Nga ekobali nibanani? | *Select all that apply:* 00 People you live with 01 Relatives you do not live with  02 People at school 03 Healthcare workers 04 Other 888 Refused to answer  *Sala fyonse ifilingile:* 00 Abantu wikala nabo 01 Ba lupwa abo tawikala nabo  02 Abantu baku sukulu 03 Aba bonfi abalolekesha pafya bumi 04 Bambi  888 Akana ukwasuka | *[Read Responses]* |

| **Assessing youth clinic interaction / Ukulolekesha kwakwapana kwamisepela pacipatala** | | | |
| --- | --- | --- | --- |
|  | How many youth at the clinic are your friends who you talk with?  Nibanga imisepela pa Cipatala aba nobe ulanda nabo? | [_________]  0-100  888 Refused to answer  0-100  888 Akana ukwasuka | *If “0”, skip to Q62.* |
|  | Do you interact with them at the clinic, outside the clinic, or both?  Bushe ulashimika nabo pa Cipatala , kunse ya Cipatala nangula konse | 01 Clinic only 02 Both at the clinic and outside the clinic 03 Outside the clinic only  888 Refused to answer  01 pa Cipatala fye  02 Konse ku Cipatala na kunse ya Cipatala 03 Kunse ya Cipatalafye  888 Akana ukwasuka |  |
|  | How close would you say you are to other youth at this clinic?  Bushe kwishibana kwamusangonshi wakwata nemisepela shimbi pano pa Cipatala? | 00 Not close at all 01 Somewhat close 02 Very close  888 Refused to answer  00 nshaba beleshapo 01 panonofye 02 sanafye  888 akana ukwasuka |  |
|  | How often do you talk with other youth at the clinic about living with HIV?  Miku inga ulanda nemisepela shimbi pa kwikala nakashishi ka HIV? | 00 Never 01 Rarely 02 Sometimes 03 Often  888 Refused to answer  00 Awe 01 Patali patali 02 Limolimo 03 lyonsefye  888 Akana kwasuka |  |
|  | Have you ever been part of a DREAMS activity, such as [add name of DREAMS activities in Ndola]?  Bushe walitala sangwamo mukufwailikisha kwaba Dreams ifyapala (ishina lya dreams mu Ndola) | 00 No  01 Yes  777 Don’t know  888 Refused to answer  00 Awe  01 Emukwai  777 Teshibe  888 Akana ukwasuka |  |
|  | If yes, how many times in the past 3 months were you involved?  Nga walisangwamo, miku inga mumyeshi itatu yapita? | [___]  Range 1-93  777 Don’t know  888 Refused to answer  Ukufuma 1-93  777 Teshibe  888 Akana ukwasuka |  |
| **Internalized Stigma and Stigma by association / Ukusekwa kobe noku sekwa kwabanobe** | | | |
| *Now we will ask you how you feel about your HIV status. These questions may make you feel sad. You do not have to answer any questions you do not want to. Please remember that your answers will be kept confidential. Please answer honestly as your responses will help us greatly. Please tell us if you agree or disagree with the following statements.*  *Nomba twalamipushako efyo munfwa palwa kuba naka shishi ka HIV. Aya amepusho yalalenga ukuti munfwe ububi tamulingile ukwasuka ilipusho ilyo tamulefwaya ukwasuka. Mwebukishe ukutila amasuko muletupela yakasungwa munkama. Twapapata mwasuke muli bucishinka twalatotela ngashi. Twapapata mutwebe ngamulesuminisha nangula mulekana.* | | | |
|  | *Please tell me if you agree or disagree with the following statements: Mukwai unjebe nga ulesuminisha nangu taulesuminisha pa lifi ifikonkelepo?*  It is difficult to tell other people about your HIV positive status.  Tacayanguka ukweba abantu ati nali kwata aka shishi ka HIV | 00 Disagree 01 Agree 888 Refused to answer  00 Nakana 01 Nasumina  888 akana ukwasuka |  |
|  | You don't let others know your HIV status.  Taufwaya bambi ukwishiba ati walikwata akashishi ka HIV | 00 Disagree 01 Agree 888 Refused to answer  00 Nakana 01 Nasumina  888 akana ukwasuka |  |
|  | You feel guilty that you are HIV positive  Ulomfwa kwati walilufyanya ifi wakwata aka shishi ka HIV | 00 Disagree 01 Agree 888 Refused to answer  00 Nakana 01 nasumina  888 akana ukwasuka |  |
|  | You are ashamed that you are HIV positive  Ulomfwa insoni ifi wakwata aka shishi ka HIV. | 00 Disagree 01 Agree 888 Refused to answer  00 Nakana 01 nasumina  888 akana ukwasuka |  |
|  | You sometimes feel worthless because you are HIV positive.  Limolimo ulonfya kwati uliwacabecabefye pantu walikwata aka shishi ka HIV | 00 Disagree 01 Agree 888 Refused to answer  00 Nakana 01 Nasumina  888 Akana ukwasuka |  |
| **Alcohol Use / Ukubonfya Ubwalwa** | | | |
| Survey administrator reads: *Now we will ask you some questions about drinking alcohol. Please remember we will keep your information confidential.*  *Umukalamba waku fwailikisha abelenga: nomba twala mipushako amepusho yamo yamo ayapakunwa ubwalwa. Twapapata mwibukishe kutila fyonse filefuma mukulashanya uku fika sugwa munkama.* | | | |
|  | How often do you have a drink containing alcohol?  Miku inga unwa ifyabamo ubwalwa? | 00 Never  01 Monthly or less  02 2-4 times a month  03 2-3 times a week  04 4 or more times a week  888 Refused to answer  00 Nshanwapo 01 Pa mulungu Pa mweshifye 02 imiku yibili nangu 4 mumweshi 03 imiku ibili nangu itatu mumulungu 04 imiku 4 mumulungu  88 akana ukwasuka |  |
|  | How many standard drinks containing alcohol do you have on a typical day?  Niyanga eyo twingalanda ama drink ayakwatamo ubwalwa eyo unwa pa bushiku bumo? | 00 0 drinks  01 1-2 drinks  02 3-4 drinks 03 5-6 drinks  04 7-9 drinks  05 10 or more drinks  888 Refused to answer  00 0 drinks 01 imo nangu yabili 02 yatatu nangu 4 03 5 nangu 6 04 7 nangu 9 05 10 ukuya pamulu  888 akana ukwasuka |  |
|  | How often do you have 6 or more drinks on one occasion?  Miku inga elyo unwa amabotolo 6 nangu aya cililepo ku ciila pamo? | 00 Never  01 Less than monthly  02 Monthly  03 Weekly  04 Daily or almost daily  888 Refused to answer  00 takwaba 01 umweshi taukwana 02 pa mweshi 03 pa mulungu 04 Cilabushiko nangula lyonse lyonse  888 akana ukwasuka |  |
| **Depression / Amalangulushi** | | | |
| Survey Administrator reads: *Now I would like to know how you have been feeling in the past week. Please respond by rating not at all, a little, quite a bit, and extremely. Again, these questions may make you feel anxious or sad. You do not have to answer any questions you do not want to answer.*  *Umukalamba waku fwailikisha abelenga:- pali ino inshita twatemwa ukuishiba efyo mwaleufwa pa mulungu wapiti. Twapapata mwasuke ukulingana nokupima kwenu, awe telyose panono, panono sana, sanafye, akana ukwasuka. Nakabili aya amepusho yalamyunfwisha umwenso nangula ukufulwa. Tamulingile ukwasuka amepusho ayo tamulefwaya ukwasuka.* | | | |
|  | In the past week, have you been… feeling low in energy or slowed down?  Uyu umulungu wapita bushe mwaleumfwa kwati amaka yalepwa nangu ukumfwa ukunaka? | 01 Not at all 02 A little 03 Quite a bit 04 Extremely 888 Refused to answer  *01 Awe telyose*  *02 Panono,*  *03 Panono sana,*  *04 Sanafye,*  *888 Akana kwasuka* |  |
|  | In the past week, have you been… blaming yourself for things?  uyu umulungu wapita bushe waleumfwa kwati naulufyanya pa fintu fimo? | 01 Not at all 02 A little 03 Quite a bit 04 Extremely 888 Refused to answer  *01 Awe telyose*  *02 Panono,*  *03 Panono sana,*  *04 Sanafye,*  *888 Akana kwasuka* |  |
|  | In the past week, have you been… crying easily?  uyu umulungu wapita bushe walelila icililelile? | 01 Not at all 02 A little 03 Quite a bit 04 Extremely 888 Refused to answer  *01 Awe telyose*  *02 Panono,*  *03 Panono sana,*  *04 Sanafye,*  *888 Akana kwasuka* |  |
|  | In the past week, have you been… feeling fidgety or restless?  uyu umulungu wapita, bushe waleunfwa ukupukapuka nangu ukufilwa ukutusha? | 01 Not at all 02 A little 03 Quite a bit 04 Extremely 888 Refused to answer  *01 Awe telyose*  *02 Panono,*  *03 Panono sana,*  *04 Sanafye,*  *888 Akana kwasuka* |  |
|  | In the past week, have you … had a poor appetite?  uyu umulungu wapita bushe  walefilwa ukulya? | 01 Not at all 02 A little 03 Quite a bit 04 Extremely 888 Refused to answer  *01 Awe telyose*  *02 Panono,*  *03 Panono sana,*  *04 Sanafye,*  *888 Akana kwasuka* |  |
|  | In the past week, have you … had difficulty falling asleep?  uyu umulungu wapita, bushe walefilwa ukusendama? | 01 Not at all 02 A little 03 Quite a bit 04 Extremely 888 Refused to answer  *01 Awe telyose*  *02 Panono,*  *03 Panono sana,*  *04 Sanafye,*  *888 Akana kwasuka* |  |
|  | In the past week, have you been… feeling hopeless about the future?  uyu umulungu wapita,bushe waleumfwa kwati takuli isubilo ku ntanshi yobe? | 01 Not at all 02 A little 03 Quite a bit 04 Extremely 888 Refused to answer  *01 Awe telyose*  *02 Panono,*  *03 Panono sana,*  *04 Sanafye,*  *888 Akana kwasuka* |  |
|  | In the past week, have you… been feeling sad?  uyu umulungu wapita,bushe waleunfwako ubulanda? | 01 Not at all 02 A little 03 Quite a bit 04 Extremely 888 Refused to answer  *01 Awe telyose*  *02 Panono,*  *03 Panono sana,*  *04 Sanafye,*  *888 Akana kwasuka* |  |
|  | In the past week, have you… been feeling lonely?  uyu mulungu wapita,bushe waleumfwa kwati tapali aba kutemwa wabafye weka? | 01 Not at all 02 A little 03 Quite a bit 04 Extremely 888 Refused to answer  *01 Awe telyose*  *02 Panono,*  *03 Panono sana,*  *04 Sanafye,*  *888 Akana kwasuka* |  |
|  | In the past week, have you… had thoughts about ending your life?  uyu umulungu wapita,bushe waletontonkanyapo ukuipaya? | 01 Not at all 02 A little 03 Quite a bit 04 Extremely 888 Refused to answer  *01 Awe telyose*  *02 Panono,*  *03 Panono sana,*  *04 Sanafye,*  *888 Akana kwasuka* | *If “A little”, “Quite a lot”, or “Extremely”, triggers message: “Please mark THOUGHTS OF SUICIDE IN PAST WEEK on the Referral Form (complete full Referral form at end fo survey).”* |
|  | In the past week, have you been… worrying too much about things?  Uyu umulungu wapita, bushe  walesakamana saana pafintu ifingi? | 01 Not at all 02 A little 03 Quite a bit 04 Extremely 888 Refused to answer  *01 Awe telyose*  *02 Panono,*  *03 Panono sana,*  *04 Sanafye,*  *888 Akana kwasuka* |  |
|  | In the past week, have you been… feeling no interest in things?  Uyu umulungu wapita ,Bushe waleumfwa kwati tapali ifili fyonse ifilekusekesha? | 01 Not at all 02 A little 03 Quite a bit 04 Extremely 888 Refused to answer  *01 Awe telyose*  *02 Panono,*  *03 Panono sana,*  *04 Sanafye,*  *888 Akana kwasuka* |  |
|  | In the past week, have you been… feeling that everything was an effort?  Uyu mulungu wapita,bushe waleumfwa kwati fyonse kwee shelela? | 01 Not at all 02 A little 03 Quite a bit 04 Extremely 888 Refused to answer  *01 Awe telyose*  *02 Panono,*  *03 Panono sana,*  *04 Sanafye,*  *888 Akana kwasuka* |  |
|  | In the past week, have you been… feeling worthless?  Uyu mulungu wapita, bushe waleunfwapo kwati tapali efyo uli? | 01 Not at all 02 A little 03 Quite a bit 04 Extremely 888 Refused to answer  *01 Awe telyose*  *02 Panono,*  *03 Panono sana,*  *04 Sanafye,*  *888 Akana kwasuka* |  |
|  | In the past week, have you … had loss of sexual interest or sexual pleasure?  Uyu umulungu wapita,bushe walilekele ukumfwa icilaka cakupanga icupo? | 01 Not at all 02 A little 03 Quite a bit 04 Extremely 888 Refused to answer  *01 Awe telyose*  *02 Panono,*  *03 Panono sana,*  *04 Sanafye,*  *888 Akana kwasuka* |  |
| **Sexual Intercourse Experience / Ukupitamo Ukupange icupo** | | | |
| Survey administrator reads*:* *Now I’d like to ask you about your experiences having sex. Remember this information will be kept secret and you can choose not to answer any question you don’t want to answer.*  *Umukalamba waku fwailikisha abelenga:- Nomba twala mipusha pamiunfwile yakumpanga icupo. Ibukisheni ukutila efyo mwalatweba fikasungwa munkama. Kabili kutimwasala ilipusho elyo tamulefwaya ukwasuka.* | | | |
|  | Have you ever had sex?  By sex, I mean when a male puts his penis inside of a female’s vagina  Bushe mwalitala pangapo icupo? Icupo ndepilibula ukutila umwaume abula ubwamba bwakwe abika mukati mu bwamba bwa mwanakashi? | 00 No, I have never had sex. 01 Yes, I have had sex. 777 Don’t know 888 Refused to answer  00 Awe shapangapo icupo  01 Emukwai nalipangapo icupo  777 Teshibe  888 Akana kwasuka | *If NO, skip to Q93.*  *If YES, proceed to next question.* |
|  | Have you had sex more than one time?  Bushe walitala pangapo ichupo ukucila pamuku umo? | 00 No, have only had sex once 01 Yes 777 Don’t know 888 Refused to answer  00 Awe na pangapofye umuku umo  01 Emukwai  777 Teshibe  888 Akana kwasuka |  |
|  | In total, with how many people have you ever had sex with?  Bonsefye, nibanga abantu wa panga nabo icupo? | 01 1 02 2 03 3 04 4 05 5 or more 777 Don’t know 888 Refused to answer  01 1 02 2 03 3 04 4 05 Ukucilapo pali 5  777 Teshibe  888 Akana ukwasuka |  |
|  | Did you use a condom the last time you had sex?  Iya kulekelesha ukupanga icupo,bushe wali bomfyeshe umupila nangu? | 00 No  01 Yes 777 Don’t remember/Don’t know 888 Refused to answer  00 Awe 01 Emukwai  777 Teti bukeshe/Teshibe  888 Akana ukwasuka |  |
| **Health Communication Self-efficacy / Ukusabakanya kwabumi palwaine** | | | |
| Survey administrator reads*: Now we want to know how confident you are that you can do things that will help keep you safe from AIDS and pregnancy. We would like you to answer these questions saying that you are very confident, a little confident, not confident. If you have not had sex, then think about how you would feel about these questions in the future when you start having sex.*  *Umukalamba waku fwailikisha abelenga:- nomba tulefwaya ukwishiba ukukosa kobe palwa fintu ifinga fwilisha ukuitalusha kuli AIDS ne fumo. Kuti twatemwa ukutila mwayasuka amepusho aya Mukukosa, Ukukosako panono uku kana konse. Nga tawatala pangapo icupo ninshi mwalamba uku totokanya efyo mwingonfwa pali aya amepusho yakuntanshi yenu elyo mukaamba ukupanga icupo.* | | | |
|  | How confident are you that you can talk to a healthcare provider about ways to prevent pregnancy?  Bushe cicetekelo cashani wakwata icakulanda naba bomfi abalolekesha pafya bumi pafya kuicingilila uku kana kwata ifumo? | 00 Not confident 01 A little confident 02 Very confident 888 Refused to answer  00 Shakosa  01 Kukosako panono  02 Kukosa sana  888 Akana kwasuka |  |
|  | If you want to have a baby in the future, how confident are you that you can talk to a healthcare provider about ways to reduce the chance of a baby getting HIV?  Nga ulefwaya ukukwata umwana kuntanshi, Bushe kuti wakwata ukokosa kwa shani pakulanda na babonfi balolekesha pafya bubi palwa kucefya ukwambukila kumwana naka shishi ka HIV? | 00 Not confident 01 A little confident 02 Very confident 888 Refused to answer  00 Shakosa  01 Kukosako panono  02 Kukosa sana  888 Akana kwasuka |  |
|  | How confident do you feel that you would tell a sexual partner about your HIV status?  Bushe kukosa kwashani wingakwata pamutemwikwa obe uwo mwanpana nankwe palwa kupanga icupo ati walikwata aka shishi ka HIV? | 00 Not confident 01 A little confident 02 Very confident 888 Refused to answer  00 Shakosa  01 Kukosako panono  02 Kukosa sana  888 Akana kwasuka |  |
|  | How confident do you feel that you would ask a sexual partner about their HIV status?  Bushe kukosa kwashani mwigonfya pakwipusha umutemwikwa obe uwo mwapana nankwe pali bena ukukwata aka shishi ka HIV? | 00 Not confident 01 A little confident 02 Very confident 888 Refused to answer  00 Shakosa  01 Kukosako panono  02 Kukosa sana  888 Akana kwasuka |  |
| **Beliefs About the Future / Ukucetekela fyakutanshi** | | | |
| Survey administrator reads: *Now we are going to ask you some questions about how you feel about your future. Please tell us if you agree or disagree with the following statements*.  *Umukalamba waku fwailikisha abelenga:-nomba twalamipusha amepusho pafyo munfwa pafya kutanshi yenu. Twapapata mutwebe nga mulesuminisha nangula mulekana pali aya amepusho aya konkelepo.* | | | |
|  | You are willing to sacrifice your immediate happiness for things that you expect to happen in the future  uli uwaipelesha ukusha efyo ukabila pali inonshita ku fintu ifinga kusansamusha kuntanshi yobe. | 00 Disagree 01 Agree  777 Don’t know 888 Refused to answer  00 Nakana  01 Nasumina  777 teshibe  888 Akana ukwasuka |  |
|  | You believe that you are in control of what will happen to you in the future  Ulecetekela ukuti watungulula fyonse ifinga kucitikila kuntanshi. | 00 Disagree 01 Agree  777 Don’t know 888 Refused to answer  00 Nakana  01 Nasumina  777 teshibe  888 Akana ukwasuka |  |
|  | **In the future**, you think you can have your own children if you want to.  Kuntashi ,bushe ulatontonkanya ukukwata abana abobe nga watemwa | 00 Disagree 01 Agree  777 Don’t know 888 Refused to answer  00 Nakana  01 Nasumina  777 teshibe  888 Akana ukwasuka |  |
|  | **In the future**, you think you will be able to get a job that you like.  kuntanshi bushe ulatontonkanya ukukwata incito iyo watemwa | 00 Disagree 01 Agree  777 Don’t know 888 Refused to answer  00 Nakana  01 Nasumina  777 teshibe  888 Akana ukwasuka |  |
|  | **In the future,** you believe if you take your ART you will be healthy.  Kuntanshi bushe walisumina ukuti nga wanwa umuti ukakwata ubumi ubusuma | 00 Disagree 01 Agree  777 Don’t know 888 Refused to answer  00 Nakana  01 Nasumina  777 teshibe  888 Akana ukwasuka |  |
| **ART Adherence /Ukukonka iminwine yamuti** | | | |
| *Now we will ask you three questions about whether you have missed taking any ART tablets. Remember that your answers will be confidential*  *Umukalamba waku fwailikisha abelenga:-nomba twalamipusha amepusho yatatu aya kutila bushe mwalitala pishapo ukunwa umuti? Mwibukishe ukutila amasuko yenu yakasugwa munkama* | | | |
|  | In the last 30 days, on how many days did you miss at least one dose of any of your ART drugs?  Pa nshiku 30 shapitile, bushe wapushilepo inshiku shinga ukunwa umuti wama ARVs? | [ __ __ ] days (0-30)  888 Refused to answer  (____) inshiku (0-30)  888 Akana kwasuka |  |
|  | In the last 30 days, how good a job did you do at taking your ART drugs in the way that you were supposed to?  Pa nshinku 30 ishipitile, wabombele shani muminwine yamuti mukukonka nefyo ulingile ukunwa? | 01 Very poor  02 Poor  03 Fair  04 Good 05 Very good 06 Excellent  888 Refused to answer  00 Shabombele bwino sanafye  01 shabombele bwino  02 Nali bombelefye panono  03 Nali bombele bwioo  04 Nali bombele bwino sana  05 Nali bombele bwino sanafye  888 Akana Kwasuka |  |
|  | In the last 30 days, how often did you take your ART drugs in the way that you were supposed to?  Inshiku shapitapo 30, imiku inga wanwinepo umuti efyo ulingile ukunwa | 01 Never  02 Sometimes  03 Often  04 Always  888 Refused to answer  01 Awe Nakalya  02 Limo limo  03 lyonsefye  04 Sanafye  888 Akana ukwasuka |  |
|  | In the past three months, did you have a day when you did not take any ART drugs?   (Meaning, you did not take any ART drugs that day)  Pamyeshi itatu yapitapo, bushe kwaliko ubushiku ubo tawanwine umuti wama ARV?  (cilepiliula, tawanwine umuti wama ARV pali bulya ubushiku) | 00 No 01 Yes 777 Don’t Know 888 Refused to answer  00 Awe 01 Emukwa  777 Teshibe  888 Akana ukwasuka |  |
|  | What were the most days in a row that you missed swallowing your drugs in the past three months? A study team member can help you to estimate.  Ninshiku shinga esho wakonkenyepo ukupusa ukunwa umuti mu myeshi itatu iyapitapo?  ***Umubomfi wa ukukusambilila kuti akwafwa ukutunganya*** | [ __ __ ] days (1-93)    Record  777 for Don’t know or  888 for Refused to answer.  [__ __] ishiku (1-93)    Lebeni  777 Teshibe  888 Akana ukwasuka |  |
| **HIV Self-Management / Kuitangata weka paka shishi ka HIV** | | | |
| Survey administrator reads: *We will now ask you some questions about how you feel about doing certain things to manage your HIV. Again, there are no right or wrong answers. Everything you say is confidential.*  *Umukalamba waku fwailikisha abelenga: - Nomba twalamipusha amepusho yamo pafyo munfwa nga mulecita ifintu fimo palwa kuitangata pa kashishi kenu ka HIV. Nakabili, takuli ubwasuko ububi nangula ubutuntulu. fyonse ifyo mwalalanda tulefisunga mukama.* | | | |
|  | In the past month, how much confidence or trust did you have to take your ART drugs even when they made you uncomfortable, unwell, or started disturbing your daily activities?  Mu mweshi wapita, bushe waicetekele shani ilyo walenwa umuti wa ma ARV na lintu walengele waumfwa ububi nangu ukulwalilako no kutampa ukukufulunganishisha imibombele yobe iya lyonsefye. | 01 Not at all confident 02 A little confident 03 Mostly confident 04 Completely confident  888 Refused to answer  01 Shakwete nokukosa sana  02 Nali koselekofye panono  03 Nalikoseleko sanafye  04 nalifye uwapwililika mukokosa  888 Akana kwasuka |  |
|  | In the past month, how much confidence or trust did you have to make taking your ART drugs part of your daily routine -- something you do every day?  Mu mweshi wapita, bushe wakosele shani nokuicetekela pa kunwa umuti wa ma ARV cila bushiku- ifitu ucita cila ubushiku? | 01 Not at all confident 02 A little confident 03 Mostly confident 04 Completely confident  888 Refused to answer  01 Shakwete nokukosa sana  02 Nali koselekofye panono  03 Nalikoseleko sanafye  04 nalifye uwapwililika mukokosa  888 Akana kwasuka |  |
|  | In the past month, how much confidence or trust did you have to take your ART drugs as part of your daily routine even if it means taking the drugs in front of people who don't know your HIV-positive status?  Mu mweshi wapita, bushe wakosele shani nokuicetekela pa kunwa umuti wa ma ARV cila bushiku nangula ulenwa pa bantu bambi aba shaishiba ukuti walikwata HIV? | 01 Not at all confident 02 A little confident 03 Mostly confident 04 Completely confident  888 Refused to answer  01 Shakwete nokukosa sana  02 Nali koselekofye panono  03 Nalikoseleko sanafye  04 nalifye uwapwililika mukokosa  888 Akana kwasuka |  |
|  | In the past month, how much confidence or trust did you have to take your ART drugs correctly even when your daily routine is disrupted?  Mu mweshi wapita, bushe wakosele shani nokui cetekela pa kunwa umuti wa ARV filya fine fye ufwile ukukonka pa kunwa nangu ca kuti calipumfyenye ifyo ucita we mwine cila bushiku? | 01 Not at all confident 02 A little confident 03 Mostly confident 04 Completely confident  888 Refused to answer  01 Shakwete nokukosa sana  02 Nali koselekofye panono  03 Nalikoseleko sanafye  04 nalifye uwapwililika mukokosa  888 Akana kwasuka |  |
|  | In the past month, how much confidence or trust did you have to take your ART drugs correctly when you are not feeling well?  Mu mweshi wapita, bushe wakosele shani nokuicetekela pa kunwa umuti wa ma ARV filya fine ufwile ukukonka pa kunwa na lintu uleumfwa ukulwala? | 01 Not at all confident 02 A little confident 03 Mostly confident 04 Completely confident  888 Refused to answer  01 Shakwete nokukosa sana  02 Nali koselekofye panono  03 Nalikoseleko sanafye  04 nalifye uwapwililika mukokosa  888 Akana kwasuka |  |
|  | In the past month, how much confidence or trust did you have to take your ART drugs even when it means changing your eating habits?  Mu mweshi wapita, bushe wakosele shani nokuicetekela pa kunwa umuti wa ma ARV nangula ca kuti walicinjishe ne milile ya fya kulya? | 01 Not at all confident 02 A little confident 03 Mostly confident 04 Completely confident  888 Refused to answer  01 Shakwete nokukosa sana  02 Nali koselekofye panono  03 Nalikoseleko sanafye  04 nalifye uwapwililika mukokosa  888 Akana kwasuka |  |
|  | In the past month, how much confidence or trust did you have to continue taking your ART drugs even if it interferes with your daily activities?  Mu mweshi wapita, bushe wakosele shani noku icetekela pa kunwa umuti wa ma ARV nangu ca kuti capumfyanya ifintu ifyo ucita cila bushiku? | 01 Not at all confident 02 A little confident 03 Mostly confident 04 Completely confident  888 Refused to answer  01 Shakwete nokukosa sana  02 Nali koselekofye panono  03 Nalikoseleko sanafye  04 nalifye uwapwililika mukokosa  888 Akana kwasuka |  |
|  | In the past month, how much confidence or trust did you have to continue taking your ART drugs when you are feeling discouraged about your health?  Mu mweshi wapita, bushe wakosele shani nokuicetekela pa kunwa umuti wa ma ARV na lintu umfwa ukunenuka pa mulandu wa bumi bobe? | 01 Not at all confident 02 A little confident 03 Mostly confident 04 Completely confident  888 Refused to answer  01 Shakwete nokukosa sana  02 Nali koselekofye panono  03 Nalikoseleko sanafye  04 nalifye uwapwililika mukokosa  888 Akana kwasuka |  |
|  | In the past month, how much confidence or trust did you have to continue taking your ART drugs even when getting to your clinic appointment is a major problem?  Mu mweshi wapita, bushe wakosele shani nokuicetekela pa kokokanya ukunwa umuti wa ma ARV nangula cashupile pakuya ku cipatala kuku-kumona? | 01 Not at all confident 02 A little confident 03 Mostly confident 04 Completely confident  888 Refused to answer  01 Shakwete nokukosa sana  02 Nali koselekofye panono  03 Nalikoseleko sanafye  04 nalifye uwapwililika mukokosa  888 Akana kwasuka |  |
|  | In the past month, how much confidence or trust did you have to continue taking your ART drugs even when people close to you tell you that they don't think it is helping?  Mu mweshi wapita, bushe wakosele shani nokuicetekela pakukonkanyapo ukunwa umuti wa ma ARV nangu ca kuti abena mupalamano bakweba ati umuti tapali ifyo ulekwafwa? | 01 Not at all confident 02 A little confident 03 Mostly confident 04 Completely confident  888 Refused to answer  01 Shakwete nokukosa sana  02 Nali koselekofye panono  03 Nalikoseleko sanafye  04 nalifye uwapwililika mukokosa  888 Akana kwasuka |  |
| Witnessing Violence / Ite yafyongo | | | |
|  | Have you ever seen or overheard your father/male guardian hit or beat your mother/female guardian?  Bushe walimonapo nangu ukunfya abafyashi abaume/ abaume bakusunga baleuma abafyashi abanakashi/abanakashi bakusunga? | 00 Never  01 Once  02 A few times  03 Many times  888 Refused to answer  00 Awe  01 Muku umo  02 Limolimo  03 Imiku Iyingi  888 Akana kwasuka |  |
| **Attitudes towards violence / Imisango yitwala kufyongo** | | | |
| *Now I will ask you some questions about what you feel is acceptable regarding men and women. Please indicate if you agree, partially agree, or do not agree. Once again, your responses remain confidential.*  *Umukalamba waku fwailikisha abelenga:-* Nomba nalamipushako amepusho efyo munfwa ifyasuminishiwa pa baume na banakashi. Twapapata mutwebe nga mulesuminisha, mulesuminisha panono nangula mulekana. Nakabali ukwasuka kwenu kulebafye kwamunkama.. | | | |
|  | There are times when a woman deserves to be beaten.  Kwaliba shimo inshita elyo umwanakashi afwaikwa ukumumaa. | 01 Agree  02 Partially agree  03 Do not agree  888 Refused to answer  01 Nasumina  02 Nasuminafye panono  03 Shile suminisha  888 Akana kwasuka |  |
|  | A woman should obey her boyfriend/husband in all things.  Umwanakashi afwile akwata icunfwila ku balume bakwe muli fyonse ifintu. | 01 Agree  02 Partially agree  03 Do not agree  888 Refused to answer  01 Nasumina  02 Nasuminafye panono  03 Shile suminisha  888 Akana kwasuka |  |
|  | Sometimes a man needs to put a woman in her place.  Shimo inshita umwaume afwile ukubika umwanakashi muncenda alefwaikwa | 01 Agree  02 Partially agree  03 Do not agree  888 Refused to answer  01 Nasumina  02 Nasuminafye panono  03 Shile suminisha  888 Akana kwasuka |  |
|  | A man using violence against his girlfriend or wife is a private matter that shouldn’t be discussed outside the couple.  Umwaume kubomfya ubukangalume kuli girlfriend nangu umukashi wakwe, lyashi lya mu ng’anda ilishilingile ukufuma kunse ya cupo | 01 Agree  02 Partially agree  03 Do not agree  888 Refused to answer  01 Nasumina  02 Nasuminafye panono  03 Shile suminisha  888 Akana kwasuka |  |
|  | A woman should tolerate violence to keep her family together.  Umwanakashi afwile ukusuminisha ubukangalume pakuti asunge ulupwa pamo | 01 Agree  02 Partially agree  03 Do not agree  888 Refused to answer  01 Nasumina  02 Nasuminafye panono  03 Shile suminisha  888 Akana kwasuka |  |
|  | A man should have the final word about decisions in his home.  Umwaume afwile akwata ishiwi ilyakulekelesha mu ng’anda yakwe. | 01 Agree  02 Partially agree  03 Do not agree  888 Refused to answer  01 Nasumina  02 Nasuminafye panono  03 Shile suminisha  888 Akana kwasuka |  |
|  | A man is expected to discipline his woman.  Umwaume alingile uku funda umwanakashi wakwe. | 01 Agree  02 Partially agree  03 Do not agree  888 Refused to answer  01 Nasumina  02 Nasuminafye panono  03 Shile suminisha  888 Akana kwasuka |  |
|  | A woman who is unfaithful needs to be put in her place.  Umwanakashi uwule cita ubucende afwile uku fundwa nokumubika muncende yakwe | 01 Agree  02 Partially agree  03 Do not agree  888 Refused to answer  01 Nasumina  02 Nasuminafye panono  03 Shile suminisha  888 Akana kwasuka |  |

| **Experiencing Violence – Emotional, Physical, Sexual / Mukupita mufyongo/imyunfwile, Kumubili, Mukupanga icupo** | | | | |
| --- | --- | --- | --- | --- |
| *Young men and women all over the world may experience violence from strangers but also from people they know well, such as a romantic partner, teacher, or family member. We are not referring to things that might happen when you are playing or having fun with your peers. The next questions are personal and could be uncomfortable to answer. Remember that you can skip any questions that you would prefer not to answer. The following questions will include things that can happen to young men and women from a range of people, including a romantic partner. By romantic partner, I mean a boyfriend or girlfriend, fiancé, or husband or wife.*  *Abalumendo naba kashana mucalo conse bali pitamo mufyongo ukufuma kubeni kabili nakubantu ebo bashiba bwino bwino, ifilinga abatemwikwa babo, baka fundisha nabantu bamulupwa wabo. Tatuletwala kufintu ifingacitika elyo bale yangala nangula elyo balesangala nabanabo. Amepusho ayakokapo yapalwenu kabilikuti yalenga imwe ukukana ufwa bwino pakwasuka. Ibukisheni ukutila kuti mwatoloka amepusho eyo tamulefwaya ukwasuka. Amepusho yakonkelepo yali mufintu ifingacitika kuba lumendo naba kashana kuli abo ababa mucitemwiko. Aba temwikwa ndepilibula ukutila boyfriend na girlfriend ,, abakobekela, Abalume naba kasha.* | | | | |
| Emotional violence/ Imyufwile ya fyongo | | | | |
| **Q** | **Has anyone ever:**  **Bushe kuliko umo uwacitapo ifi:** | **A)**  **Yes=01**  **No=00**  **Emukwai =01**  **Awe =02** | **B) How often has this happened in the past 12 months?**  Fyacitika imiku inga pamyeshi 12 yapitapo? | **C) Who did this to you in the past 12 months?**  **Nibani bakucitile ifi mu myeshi 12 yapitapo?**  *Survey administrator should read each answer option individually. Participant should select all that apply.* |
|  | Insulted you or made you feel bad about yourself?  Balikutukile nangula balileka iwe ukunfwa ububi? | 00 No  01 Yes  888 Refused to answer  00 Awe 01 Emukwai  888 Akana ukwasuka  *If NO, skip to Q127.*  *If YES, proceed to Q126b.* | 00 Never  01 Once  02 A few times  03 Many times  888 Refused to answer  00 Awe  01 Muku umo  02 Limo Limo  03 inshita Iyingi  888 Akana Kwasuka  *If Never, skip to Q127.*  *Otherwise, proceed to Q126c.* | 01 Romantic partner  02 Parent/caregiver  03 Other family  Member  04 Friend or peer  05 Stranger  06 School staff member  07 Employer  08 Health care worker  09 Neighbor  10 Religious leader  11 Military/police  12 Orphanage staff  13 Someone else you know  888 Refused to answer  01 Mutemwikwa wandi  02 Abafyashi/Abasunga  03 Balupwa bambi  04 Abanandi  05 Abeni  06Aba kususkulu  07 Aba kuncito  08 Aba Bofi ba bumi  09 Abena Mpalamano  10 Intungulushi yamapepo  11 aba soja/ ba kapokola  12 Aba bonfi baku orphanage  13 umbifye waishiba  888 Akana ukwasuka |
|  | Belittled or humiliated you in front of other people?  Balikusula No kukucefya pamenso yabantu abengi | 00 No  01 Yes  888 Refused to answer  00 Awe 01 Emukwa  888 Akana ukwasuka  *If NO, skip to Q128.*  *If YES, proceed to Q127b.* | 00 Never  01 Once  02 A few times  03 Many times  888 Refused to answer  00 Awe  01 Muku umo  02 Limo Limo  03 inshita Iyingi  888 Akana Kwasuka  *If Never, skip to Q128.*  *Otherwise, proceed to Q127c.* | 01 Romantic partner  02 Parent/caregiver  03 Other family member  04 Friend or peer  05 Stranger  06 School staff member  07 Employer  08 Health care worker  09 Neighbor  10 Religious leader  11 Military/police  12 Orphanage staff  13 Someone else you know  888 Refused to answer  01 Mutemwikwa wandi  02 Abafyashi/Abasunga  03 Balupwa bambi  04 Abanandi  05 Abeni  06Aba kususkulu  07 Aba kuncito  08 Aba Bofi ba bumi  09 Abena Mpalamano  10 Intungulushi yamapepo  11 aba soja/ ba kapokola  12 Aba bonfi baku orphanage  13 umbifye waishiba  888 Akana ukwasuka |
|  | Threatened to leave or abandon you?  Balitinya ukusha nangu uku ndekelesha | 00 No  01 Yes  888 Refused to answer  00 Awe 01 Emukwai  888 Akana ukwasuka  *If NO, skip to Q129.*  *If YES, proceed to Q128b.* | 00 Never  01 Once  02 A few times  03 Many times  888 Refused to answer  00 Awe  01 Muku umo  02 Limo Limo  03 inshita Iyingi  888 Akana Kwasuka  *If Never, skip to Q129.*  *Otherwise, proceed to Q128c.* | 01 Romantic partner  02 Parent/caregiver  03 Other family member  04 Friend or peer  05 Stranger  06 School staff member  07 Employer  08 Health care worker  09 Neighbor  10 Religious leader  11 Military/police  12 Orphanage staff  13 Someone else you know  888 Refused to answ  01 Mutemwikwa wandi  02 Abafyashi/Abasunga  03 Balupwa bambi  04 Abanandi  05 Abeni  06Aba kususkulu  07 Aba kuncito  08 Aba Bofi ba bumi  09 Abena Mpalamano  10 Intungulushi yamapepo  11 aba soja/ ba kapokola  12 Aba bonfi baku orphanage  13 umbifye waishiba  888 Akana ukwasuka |
|  | Locked you either inside or outside of the home?  Bali njisalila mukati nangu kunse yang’anda | 00 No  01 Yes  888 Refused to answer  00 Awe 01 Emukwai  888 Akana ukwasuka  *If NO, skip to Q130.*  *If YES, proceed to Q129b.* | 00 Never  01 Once  02 A few times  03 Many times  888 Refused to answer  00 Awe  01 Muku umo  02 Limo Limo  03 inshita Iyingi  888 Akana Kwasuka  *If Never, skip to Q130.*  *Otherwise, proceed to Q129c.* | 01 Romantic partner  02 Parent/caregiver  03 Other family member  04 Friend or peer  05 Stranger  06 School staff member  07 Employer  08 Health care worker  09 Neighbor  10 Religious leader  11 Military/police  12 Orphanage staff  13 Someone else you know  888 Refused to answer  01 Mutemwikwa wandi  02 Abafyashi/Abasunga  03 Balupwa bambi  04 Abanandi  05 Abeni  06Aba kususkulu  07 Aba kuncito  08 Aba Bofi ba bumi  09 Abena Mpalamano  10 Intungulushi yamapepo  11 aba soja/ ba kapokola  12 Aba bonfi baku orphanage  13 umbifye waishiba  888 Akana ukwasuka |
|  | Threatened to invoke harmful people, ghosts, or evil spirits against you?  Balitinya ukutumina abantu ababi icibanda nangula imipashi yakowela pali ine | 00 No  01 Yes  888 Refused to answer  00 Awe 01 Emukwai  888 Akana ukwasuka  *If NO, skip to Q131.*  *If YES, proceed to Q130b.* | 00 Never  01 Once  02 A few times  03 Many times  888 Refused to answer  00 Awe  01 Muku umo  02 Limo Limo  03 inshita Iyingi  888 Akana Kwasuka  *If Never, skip to Q131.*  *Otherwise, proceed to Q130c.* | 01 Romantic partner  02 Parent/caregiver  03 Other family member  04 Friend or peer  05 Stranger  06 School staff member  07 Employer  08 Health care worker  09 Neighbor  10 Religious leader  11 Military/police  12 Orphanage staff  13 Someone else you know  888 Refused to answer  01 Mutemwikwa wandi  02 Abafyashi/Abasunga  03 Balupwa bambi  04 Abanandi  05 Abeni  06Aba kususkulu  07 Aba kuncito  08 Aba Bofi ba bumi  09 Abena Mpalamano  10 Intungulushi yamapepo  11 aba soja/ ba kapokola  12 Aba bonfi baku orphanage  13 umbifye waishiba  888 Akana ukwasuka |
|  | Referred to your skin color/ gender /religion /tribe or health problems you have in a hurtful way?  Baleloosha kukanda yandi/ukuba umwaume nangu umwanakashi/  Amapepo/umutundu/nangula pabwafya bwa bumi bwandi munshila iyabipa? | 00 No  01 Yes  888 Refused to answer  00 Awe 01 Emukwai  888 Akana ukwasuka  *If NO, skip to Q132.*  *If YES, proceed to Q131b.* | 00 Never  01 Once  02 A few times  03 Many times  888 Refused to answer  00 Awe  01 Muku umo  02 Limo Limo  03 inshita Iyingi  888 Akana Kwasuka  *If Never, skip to Q132.*  *Otherwise, proceed to Q131c.* | 01 Romantic partner  02 Parent/caregiver  03 Other family member  04 Friend or peer  05 Stranger  06 School staff member  07 Employer  08 Health care worker  09 Neighbor  10 Religious leader  11 Military/police  12 Orphanage staff  13 Someone else you know  888 Refused to answer  01 Mutemwikwa wandi  02 Abafyashi/Abasunga  03 Balupwa bambi  04 Abanandi  05 Abeni  06Aba kususkulu  07 Aba kuncito  08 Aba Bofi ba bumi  09 Abena Mpalamano  10 Intungulushi yamapepo  11 aba soja/ ba kapokola  12 Aba bonfi baku orphanage  13 umbifye waishiba  888 Akana ukwasuka |

| Physical violence  ifyongo fyakumubili | | | | |
| --- | --- | --- | --- | --- |
| **Q** | **Has anyone ever:**  **Bushe kuliko umo uwacitapo ifi:** | **A)**  **Yes=01**  **No=00**  **Emukwai =01**  **Awe =02** | **B) How often has this happened in the past 12 months?**  Fyacitika imiku inga pamyeshi 12 yapitapo? | **C) Who did this to you in the past 12 months?**  **Nibani bakucitile ifi mu myeshi 12 yapitapo?**  *Survey administrator should read each answer option individually. Participant should select all that apply.* |
|  | Slapped you or thrown something at you that could hurt you?  Balikutobapo ulupi nangula icintu icinga kucena | 00 No  01 Yes  888 Refused to answer  00 Awe 01 Emukwai  888 Akana ukwasuka  *If NO, skip to Q133.*  *If YES, proceed to Q132b.* | 00 Never  01 Once  02 A few times  03 Many times  888 Refused to answer  00 Awe  01 Muku umo  02 Limo Limo  03 ishita Iyingi  888 Akana Kwasuka  *If Never, skip to Q133.*  *Otherwise, proceed to Q132c.* | 01 Romantic partner  02 Parent/caregiver  03 Other family member  04 Friend or peer  05 Stranger  06 School staff member  07 Employer  08 Health care worker  09 Neighbor  10 Religious leader  11 Military/police  12 Orphanage staff  13 Someone else you know  888 Refused to answer  01 Mutemwikwa wandi  02 Abafyashi/Abasunga  03 Balupwa bambi  04 Abanandi  05 Abeni  06Aba kususkulu  07 Aba kuncito  08 Aba Bofi ba bumi  09 Abena Mpalamano  10 Intungulushi yamapepo  11 aba soja/ ba kapokola  12 Aba bonfi baku orphanage  13 umbifye waishiba  888 Akana ukwasuka |
|  | Pushed you or shoved you?  Balikusunkapo nagu ukukwempapo? | 00 No  01 Yes  888 Refused to answer  00 Awe 01 Emukwai  888 Akana ukwasuka  *If NO, skip to Q134.*  *If YES, proceed to Q133b.* | 00 Never  01 Once  02 A few times  03 Many times  888 Refused to answer  00 Awe  01 Muku umo  02 Limo Limo  03 ishita Iyingi  888 Akana Kwasuka  *If Never, skip to Q134.*  *Otherwise, proceed to Q133c.* | 01 Romantic partner  02 Parent/caregiver  03 Other family member  04 Friend or peer  05 Stranger  06 School staff member  07 Employer  08 Health care worker  09 Neighbor  10 Religious leader  11 Military/police  12 Orphanage staff  13 Someone else you know  888 Refused to answer  01 Mutemwikwa wandi  02 Abafyashi/Abasunga  03 Balupwa bambi  04 Abanandi  05 Abeni  06Aba kususkulu  07 Aba kuncito  08 Aba Bofi ba bumi  09 Abena Mpalamano  10 Intungulushi yamapepo  11 aba soja/ ba kapokola  12 Aba bonfi baku orphanage  13 umbifye waishiba  888 Akana ukwasuka |
|  | Twisted your ear or arm as punishment?  Balishongotula ukutwi nangulaukuboko paku panikwa? | 00 No  01 Yes  888 Refused to answer  00 Awe 01 Emukwai  888 Akana ukwasuka  *If NO, skip to Q135.*  *If YES, proceed to Q134b.* | 00 Never  01 Once  02 A few times  03 Many times  888 Refused to answer  00 Awe  01 Muku umo  02 Limo Limo  03 ishita Iyingi  888 Akana Kwasuka  *If Never, skip to Q135.*  *Otherwise, proceed to Q134c.* | 01 Romantic partner  02 Parent/caregiver  03 Other family member  04 Friend or peer  05 Stranger  06 School staff member  07 Employer  08 Health care worker  09 Neighbor  10 Religious leader  11 Military/police  12 Orphanage staff  13 Someone else you know  888 Refused to answer  01 Mutemwikwa wandi  02 Abafyashi/Abasunga  03 Balupwa bambi  04 Abanandi  05 Abeni  06Aba kususkulu  07 Aba kuncito  08 Aba Bofi ba bumi  09 Abena Mpalamano  10 Intungulushi yamapepo  11 aba soja/ ba kapokola  12 Aba bonfi baku orphanage  13 umbifye waishiba  888 Akana ukwasuka |
|  | Hit you with a fist or with something else that could hurt you, such as a stick or cane?  Balikutobapo ekofi nangula icintu cimo icinga cena, icilinga ubwebya nangula icimuti? | 00 No  01 Yes  888 Refused to answer  00 Awe 01 Emukwai  888 Akana ukwasuka  *If NO, skip to Q136.*  *If YES, proceed to Q135b.* | 00 Never  01 Once  02 A few times  03 Many times  888 Refused to answer  00 Awe  01 Muku umo  02 Limo Limo  03 ishita Iyingi  888 Akana Kwasuka  *If Never, skip to Q136.*  *Otherwise, proceed to Q135c.* | 01 Romantic partner  02 Parent/caregiver  03 Other family member  04 Friend or peer  05 Stranger  06 School staff member  07 Employer  08 Health care worker  09 Neighbor  10 Religious leader  11 Military/police  12 Orphanage staff  13 Someone else you know  888 Refused to answer  01 Mutemwikwa wandi  02 Abafyashi/Abasunga  03 Balupwa bambi  04 Abanandi  05 Abeni  06Aba kususkulu  07 Aba kuncito  08 Aba Bofi ba bumi  09 Abena Mpalamano  10 Intungulushi yamapepo  11 aba soja/ ba kapokola  12 Aba bonfi baku orphanage  13 umbifye waishiba  888 Akana ukwasuka |
|  | Kicked you, dragged you, or severely beaten you up?  *Baliku pantapo nangu ukukusunkapo nangula ukukulopolapo sana?*  *(WHO measure of severe physical violence)* | 00 No  01 Yes  888 Refused to answer  00 Awe 01 Eee  888 Akana ukwasuka  *If NO, skip to Q137.*  *If YES, proceed to Q136b.* | 00 Never  01 Once  02 A few times  03 Many times  888 Refused to answer  00 Awe  01 Muku umo  02 Limo Limo  03 ishita Iyingi  888 Akana Kwasuka  *If Never, skip to Q137.*  *If participant reports this experience “Once”, “A few times”, or “Many times” in past 12 months, proceed Q136c, then survey triggers administrative message: “Please mark this experience of SEVERE PHYSICAL VIOLENCE on the Referral Form (complete full Referral form at end fo survey).”* | 01 Romantic partner  02 Parent/caregiver  03 Other family member  04 Friend or peer  05 Stranger  06 School staff member  07 Employer  08 Health care worker  09 Neighbor  10 Religious leader  11 Military/police  12 Orphanage staff  13 Someone else you know  888 Refused to answer  01 Mutemwikwa wandi  02 Abafyashi/Abasunga  03 Balupwa bambi  04 Abanandi  05 Abeni  06Aba kususkulu  07 Aba kuncito  08 Aba Bofi ba bumi  09 Abena Mpalamano  10 Intungulushi yamapepo  11 aba soja/ ba kapokola  12 Aba bonfi baku orphanage  13 umbifye waishiba  888 Akana ukwasuka |
|  | Choked you or burnt you on purpose?  *Baliku kamapo pamukoshi nangula uku koca kumufulofye*  *(WHO measure of severe physical violence)* | 00 No  01 Yes  888 Refused to answer  00 Awe 01 Eee  888 Akana ukwasuka  *If NO, skip to Q138.*  *If YES, proceed to Q137b.* | 00 Never  01 Once  02 A few times  03 Many times  888 Refused to answer  00 Awe  01 Muku umo  02 Limo Limo  03 ishita Iyingi  888 Akana Kwasuka  *If Never, skip to Q138.*  *If participant reports this experience “Once”, “A few times”, or “Many times” in past 12 months, proceed Q137c, then survey triggers administrative message: “Please mark this experience of SEVERE PHYSICAL VIOLENCE on the Referral Form (complete full Referral form at end fo survey).”* | 01 Romantic partner  02 Parent/caregiver  03 Other family member  04 Friend or peer  05 Stranger  06 School staff member  07 Employer  08 Health care worker  09 Neighbor  10 Religious leader  11 Military/police  12 Orphanage staff  13 Someone else you know  888 Refused to answer  01 Mutemwikwa wandi  02 Abafyashi/Abasunga  03 Balupwa bambi  04 Abanandi  05 Abeni  06Aba kususkulu  07 Aba kuncito  08 Aba Bofi ba bumi  09 Abena Mpalamano  10 Intungulushi yamapepo  11 aba soja/ ba kapokola  12 Aba bonfi baku orphanage  13 umbifye waishiba  888 Akana ukwasuka |
|  | Threatened to use or actually used a sharp object or other weapon against you?  Baliku tinyapo ukubofya nangula babofyeshefye icela icatwa kuli iwe.  *(WHO measure of severe physical violence)* | 00 No  01 Yes  888 Refused to answer  00 Awe 01 Eee  888 Akana ukwasuka  *If NO, skip to Q139.*  *If YES, proceed to Q138b.* | 00 Never  01 Once  02 A few times  03 Many times  888 Refused to answer  00 Awe  01 Muku umo  02 Limo Limo  03 ishita Iyingi  888 Akana Kwasuka  *If Never, skip to Q139.*  *If participant reports this experience “Once”, “A few times”, or “Many times” in past 12 months, proceed Q138c, then survey triggers administrative message: “Please mark this experience of SEVERE PHYSICAL VIOLENCE on the Referral Form (complete full Referral form at end fo survey).”* | 01 Romantic partner  02 Parent/caregiver  03 Other family member  04 Friend or peer  05 Stranger  06 School staff member  07 Employer  08 Health care worker  09 Neighbor  10 Religious leader  11 Military/police  12 Orphanage staff  13 Someone else you know  888 Refused to answer  01 Mutemwikwa wandi  02 Abafyashi/Abasunga  03 Balupwa bambi  04 Abanandi  05 Abeni  06Aba kususkulu  07 Aba kuncito  08 Aba Bofi ba bumi  09 Abena Mpalamano  10 Intungulushi yamapepo  11 aba soja/ ba kapokola  12 Aba bonfi baku orphanage  13 umbifye waishiba  888 Akana ukwasuka |

| Sexual violence / Ukucinta Icupo kubukangalume | | | | | | | | |
| --- | --- | --- | --- | --- | --- | --- | --- | --- |
| **Q** | **Has anyone ever:**  **Bushe kuliko umo uwacitapo ifi:** | | **A)**  **Yes=01**  **No=00**  **Emukwai =01**  **Awe =02** | **B) At what age did this first occur?**  **Wakwete imyaka inga elyo fyacitike?** | | **B) How often has this happened in the past 12 months?**  Fyacitika imiku inga pamyeshi 12 yapitapo? | | **C) Who did this to you in the past 12 months?**  **Nibani ba kucitile ifi mu myeshi 12 yapitapo**  *Survey administrator should read each answer option individually. Participant should select all that apply.* |
|  | Made you watch a sex video or look at sexual pictures?  Balengele watamba ifikope nangula ifipikica ifyakupanga icupo?  *If participant reports this experience ever, triggers administrative message: “Please mark this experience of SEXUAL VIOLENCE on the Referral Form (complete full Referral form at end fo survey).”* | | 00 No  01 Yes  888 Refused to answer  00 Awe 01 Emukwai  888 Akana ukwasuka  *If NO, skip to Q140.*  *If YES, proceed to Q139b.* | Enter age: __  Lembeni imyaka | | 00 Never  01 Once  02 A few times  03 Many times  888 Refused to answer  00 Awe  01 Muku umo  02 Limo Limo  03 ishita Iyingi  888 Akana Kwasuka  *If Never, skip to Q140.*  *Otherwise, proceed to Q139c.* | | 01 Romantic partner  02 Parent/caregiver  03 Other family member  04 Friend or peer  05 Stranger  06 School staff member  07 Employer  08 Health care worker  09 Neighbor  10 Religious leader  11 Military/police  12 Orphanage staff  13 Someone else you know  888 Refused to answer  01 Mutemwikwa wandi  02 Abafyashi/Abasunga  03 Balupwa bambi  04 Abanandi  05 Abeni  06Aba kususkulu  07 Aba kuncito  08 Aba Bofi ba bumi  09 Abena Mpalamano  10 Intungulushi yamapepo  11 aba soja/ ba kapokola  12 Aba bonfi baku orphanage  13 umbifye waishiba  888 Akana ukwasuka |
|  | Made you look at their private parts or wanted to look at yours?  Balenga watamba pa bwamba bwabo nangu balefwaya ukutamba ubwamba bobe?  *If participant reports this experience ever, triggers administrative message: “Please mark this experience of SEXUAL VIOLENCE on the Referral Form (complete full Referral form at end fo survey).”* | | 00 No  01 Yes  888 Refused to answer  00 Awe 01 Emukwai  888 Akana ukwasuka  *If NO, skip to Q141.*  *If YES, proceed to Q140b.* | Enter age: __  Lembeni imyaka | | 00 Never  01 Once  02 A few times  03 Many times  888 Refused to answer  00 Awe  01 Muku umo  02 Limo Limo  03 ishita Iyingi  888 Akana Kwasuka  *If Never, skip to Q141.*  *Otherwise, proceed to Q140c.* | | 01 Romantic partner  02 Parent/caregiver  03 Other family member  04 Friend or peer  05 Stranger  06 School staff member  07 Employer  08 Health care worker  09 Neighbor  10 Religious leader  11 Military/police  12 Orphanage staff  13 Someone else you know  888 Refused to answer  01 Mutemwikwa wandi  02 Abafyashi/Abasunga  03 Balupwa bambi  04 Abanandi  05 Abeni  06Aba kususkulu  07 Aba kuncito  08 Aba Bofi ba bumi  09 Abena Mpalamano  10 Intungulushi yamapepo  11 aba soja/ ba kapokola  12 Aba bonfi baku orphanage  13 umbifye waishiba  888 Akana ukwasuka |
|  | Touched your private parts in a sexual way, or made you touch theirs?  Balikete pamwamba wobe nokulenga wikate pamwamba wabo munshila yakupange icupo?  *If participant reports this experience ever, triggers administrative message: “Please mark this experience of SEXUAL VIOLENCE on the Referral Form (complete full Referral form at end fo survey).”* | | 00 No  01 Yes  888 Refused to answer  00 Awe 01 Emukwa  888 Akana ukwasuka  *If NO, skip to Q142.*  *If YES, proceed to Q141b.* | Enter age: __  Lembeni imyaka | | 00 Never  01 Once  02 A few times  03 Many times  888 Refused to answer  00 Awe  01 Muku umo  02 Limo Limo  03 ishita Iyingi  888 Akana Kwasuka  *If Never, skip to Q142.*  *Otherwise, proceed to Q141c.* | | 01 Romantic partner  02 Parent/caregiver  03 Other family member  04 Friend or peer  05 Stranger  06 School staff member  07 Employer  08 Health care worker  09 Neighbor  10 Religious leader  11 Military/police  12 Orphanage staff  13 Someone else you know  888 Refused to answer  01 Mutemwikwa wandi  02 Abafyashi/Abasunga  03 Balupwa bambi  04 Abanandi  05 Abeni  06Aba kususkulu  07 Aba kuncito  08 Aba Bofi ba bumi  09 Abena Mpalamano  10 Intungulushi yamapepo  11 aba soja/ ba kapokola  12 Aba bonfi baku orphanage  13 umbifye waishiba  888 Akana ukwasuka |
|  | Physically forced you to have sexual intercourse when you did not want to?  Banpatikishe kuti tupange nabena icupo elyo nshalefyaya?  *If participant reports this experience ever, triggers administrative message: “Please mark this experience of SEXUAL VIOLENCE on the Referral Form (complete full Referral form at end fo survey).”* | | 00 No  01 Yes  888 Refused to answer  00 Awe 01 Emukwa  888 Akana ukwasuka  *If NO, skip to Q143.*  *If YES, proceed to Q142b.* | Enter age: __  Lembeni imyaka | | 00 Never  01 Once  02 A few times  03 Many times  888 Refused to answer  00 Awe  01 Muku umo  02 Limo Limo  03 ishita Iyingi  888 Akana Kwasuka  *If Never, skip to Q143.*  *Otherwise, proceed to Q142c.* | | 01 Romantic partner  02 Parent/caregiver  03 Other family member  04 Friend or peer  05 Stranger  06 School staff member  07 Employer  08 Health care worker  09 Neighbor  10 Religious leader  11 Military/police  12 Orphanage staff  13 Someone else you know  888 Refused to answer  01 Mutemwikwa wandi  02 Abafyashi/Abasunga  03 Balupwa bambi  04 Abanandi  05 Abeni  06Aba kususkulu  07 Aba kuncito  08 Aba Bofi ba bumi  09 Abena Mpalamano  10 Intungulushi yamapepo  11 aba soja/ ba kapokola  12 Aba bonfi baku orphanage  13 umbifye waishiba  888 Akana ukwasuka |
| **Mindfulness / Kubikako amano** | | | | | | | | |
| Survey administrator reads: *Below are statements about your everyday experiences. Please let me know how frequently or infrequently you currently have each experience.*  *Pafikokelepo fili pafyo tupitamo cilabushiku. Napapata ndefwaya ukwishiba nga nilyonsefye nangula te lyonsefye pafyo mupitamo.* | | | | | | | | |
|  | | You could be experiencing some emotion and not be conscious of it until some time later.  Kuti uleunfwa imyufwile yamubili (Insuna) ukwabulo ukuposako amano kano lilya papita akashita | | | 01 almost always 02 very frequently 03 somewhat frequently 04 somewhat infrequently 05 very infrequently 06 almost never  888 Refused to answer  01 Lyonsefye  02 Ishita Ne shita  03 Mushita  04 Limbi Mushita  05 Mushitafye  06 Shatala  888 akana Kwasuka | |  | |
|  | | You break or spill things because of carelessness, not paying attention, or thinking of something else. ulatoba no kwitila ifintu ku mulekelesha, ukwabula ukuposako amamo nangu muku tontonkanya palifimbi | | | 01 almost always 02 very frequently 03 somewhat frequently 04 somewhat infrequently 05 very infrequently 06 almost never  888 Refused to answer  01 Lyonsefye  02 Ishita Ne shita  03 Mushita  04 Limbi Mushita  05 Mushitafye  06 Shatala  888 akana Kwasuka | |  | |
|  | | You find it difficult to stay focused on what’s happening in the present.  ulacisanga ukukosa uku posako amano pafilecitika apopene. | | | 01 almost always 02 very frequently 03 somewhat frequently 04 somewhat infrequently 05 very infrequently 06 almost never  888 Refused to answer  01 Lyonsefye  02 Ishita Ne shita  03 Mushita  04 Limbi Mushita  05 Mushitafye  06 Shatala  888 akana Kwasuka | |  | |
|  | | You tend to walk quickly to get where you are going without paying attention to what you experience along the way.  ulacincila mukwenda ukwabula ukuposako amano kufile kucitikila munshila | | | 01 almost always 02 very frequently 03 somewhat frequently 04 somewhat infrequently 05 very infrequently 06 almost never  888 Refused to answer  01 Lyonsefye  02 Ishita Ne shita  03 Mushita  04 Limbi Mushita  05 Mushitafye  06 Shatala  888 akana Kwasuka | |  | |
|  | | You tend not to notice feelings of physical tension or discomfort until they really grab your attention.  ulesha mukukana umfwa ubukali no bubi pamulandu wakushiposako amano paka ba kwilusha | | | 01 almost always 02 very frequently 03 somewhat frequently 04 somewhat infrequently 05 very infrequently 06 almost never  888 Refused to answer  01 Lyonsefye  02 Ishita Ne shita  03 Mushita  04 Limbi Mushita  05 Mushitafye  06 Shatala  888 akana Kwasuka | |  | |
|  | | You forget a person’s name almost as soon as you’ve been told it for the first time.  Ulesa mukulaba ishina lya muntu nangu elo Bafuma mukukweba apopene umuku  Waku balipo | | | 01 almost always 02 very frequently 03 somewhat frequently 04 somewhat infrequently 05 very infrequently 06 almost never  888 Refused to answer  01 Lyonsefye  02 Ishita Ne shita  03 Mushita  04 Limbi Mushita  05 Mushitafye  06 Shatala  888 akana Kwasuka | |  | |
|  | | It seems you are "running on automatic" without much awareness of what you are doing  Cimoneka kwati ulebutuka ukwabula ukwishiba ico ulecita. | | | 01 almost always 02 very frequently 03 somewhat frequently 04 somewhat infrequently 05 very infrequently 06 almost never  888 Refused to answer  01 Lyonsefye  02 Ishita Ne shita  03 Mushita  04 Limbi Mushita  05 Mushitafye  06 Shatala  888 akana Kwasuka | |  | |
|  | | You rush through activities without being really attentive to them.  ulabutukila mumilimo iyaku cita ukwabula ukubikako amano. | | | 01 almost always 02 very frequently 03 somewhat frequently 04 somewhat infrequently 05 very infrequently 06 almost never  888 Refused to answer  01 Lyonsefye  02 Ishita Ne shita  03 Mushita  04 Limbi Mushita  05 Mushitafye  06 Shatala  888 akana Kwasuka | |  | |
|  | | You get so focused on the goal you want to achieve that you lose touch with what you are doing right now to get there.  ulabikako amano efyo ulefwaya ukucita lelo ulafilufya pafyo ulecita ukuti wingafikilisha. | | | 01 almost always 02 very frequently 03 somewhat frequently 04 somewhat infrequently 05 very infrequently 06 almost never  888 Refused to answer  01 Lyonsefye  02 Ishita Ne shita  03 Mushita  04 Limbi Mushita  05 Mushitafye  06 Shatala  888 akana Kwasuka | |  | |
|  | | You do jobs or tasks automatically, without being aware of what you’re doing.  ulabomba imilimo ukwabula nokwishiba ifyo ulecita. | | | 01 almost always 02 very frequently 03 somewhat frequently 04 somewhat infrequently 05 very infrequently 06 almost never  888 Refused to answer  01 Lyonsefye  02 Ishita Ne shita  03 Mushita  04 Limbi Mushita  05 Mushitafye  06 Shatala  888 akana Kwasuka | |  | |
|  | | You find yourself listening to someone with one ear, doing something else at the same time.  ulaisanga uleunfwa kumuntu nokutwi kumo, kaili nokucita ifitu fimbi panshita imo ine. | | | 01 almost always 02 very frequently 03 somewhat frequently 04 somewhat infrequently 05 very infrequently 06 almost never  888 Refused to answer  01 Lyonsefye  02 Ishita Ne shita  03 Mushita  04 Limbi Mushita  05 Mushitafye  06 Shatala  888 akana Kwasuka | |  | |
|  | | You go places on ‘automatic pilot’ and then wonder why you went there.  Ulaya kucende ukwabula ukwishiba elo waisa muku tontonkanya eco waciilako | | | 01 almost always 02 very frequently 03 somewhat frequently 04 somewhat infrequently 05 very infrequently 06 almost never  888 Refused to answer  01 Lyonsefye  02 Ishita Ne shita  03 Mushita  04 Limbi Mushita  05 Mushitafye  06 Shatala  888 akana Kwasuka | |  | |
|  | | You find yourself preoccupied with the future or the past.  Ulaisanga uletontonkanya ifitu infingi mumutwe pafyapita nefikaba kutashi. | | | 01 almost always 02 very frequently 03 somewhat frequently 04 somewhat infrequently 05 very infrequently 06 almost never  888 Refused to answer  01 Lyonsefye  02 Ishita Ne shita  03 Mushita  04 Limbi Mushita  05 Mushitafye  06 Shatala  888 akana Kwasuka | |  | |
|  | | You find yourself doing things without paying attention.  ulaisanga ulecita ifintu ukwabula ukuposako amano | | | 01 almost always 02 very frequently 03 somewhat frequently 04 somewhat infrequently 05 very infrequently 06 almost never  888 Refused to answer  01 Lyonsefye  02 Ishita Ne shita  03 Mushita  04 Limbi Mushita  05 Mushitafye  06 Shatala  888 akana Kwasuka | |  | |
|  | | You snack without being aware that you’re eating.  Ulalya ukwabula ukwishiba ati ulelya | | | 01 almost always 02 very frequently 03 somewhat frequently 04 somewhat infrequently 05 very infrequently 06 almost never  888 Refused to answer  01 Lyonsefye  02 Ishita Ne shita  03 Mushita  04 Limbi Mushita  05 Mushitafye  06 Shatala  888 akana Kwasuka | |  | |

| Annex – Safety follow-up **TO BE ASKED AT THE END OF THE SURVEY** | | | |
| --- | --- | --- | --- |
|  | *Question for administrator:*  Did this participant report experiences of severe violence or thoughts of suicide that triggered an alert to mark it on the Referral Form? | 00 No  01 Yes | *If NO, skip to END OF SURVEY.*  *If YES, proceed to follow-up question.* |
|  | *If YES, ask participant:*  Is the abuse currently happening (i.e. within the past month)?  Bushe ukushushiwa kucili kulecitika (pamweshi uyu wapiti)? | 00 No  01 Yes  999 Not applicable – participant only reported thoughts of suicide, not abuse. | *Regardless of response to this follow-up question, data collector will read Automatic Referral Script.* |
| Automatic Referral Script | | | |
| Survey Administrator reads: *Thank you for speaking with me and for being honest. What you have told me today has made me concerned about your wellbeing. I would like to take you to a healthcare provider who will ask you some other questions about your health and safety.*  *Umukalamba wakufwalikisha abelenga: Natotela pakulanda naine napakuba uwacishika efyo wajeba ushiku walelo nafikumya pabumi bobe. Kuti natemwa ukutwala pabalokesha pafya bumi alaya kwipushako pamesho yamo yamo pabumi bobe nefyakuisunga.* | | | |
| END OF SURVEY  Administrator reads: Thank you for taking the time to talk with me today. | | | |

**END OF SURVEY**
